# Modulation of the Plasma Lipidomic Profile in Piglets Fed Polar Lipid-Rich Diets

**DOI:** 10.3390/metabo14120673

**Published:** 2024-12-03

**Authors:** Rayllana Larsen, Salma Chakroun, Marie-Pierre Létourneau-Montminy, Janie Levesque, Dimas Estrasulas de Oliveira, Jorge Eduardo Rico, Daniel E. Rico

**Affiliations:** 1 Department of Animal Production, Santa Catarina State University, Lages 88.520-000, SC, Brazil; larsen.rayllana@gmail.com (R.L.);; 2 Department of Animal Science, Université Laval, Quebec, QC G1V 0A6, Canada; 3 Centre de Recherche en Sciences Animales de Deschambault, Quebec, QC G0A 1S0, Canada; 4 Department of Animal and Avian Sciences, University of Maryland, College Park, MD 20742-2311, USA; jerico@umd.edu; 5 Institute of Nutrition and Functional Foods, Université Laval, Quebec, QC G1V 0A6, Canada

**Keywords:** plant-based feed, sphingolipids, phospholipids, piglet, plasma lipidome

## Abstract

**Background:** Polar lipids from dairy are novel sources of energy that may replace other dietary lipids and impact plasma lipidomic profiles in piglets. This study evaluated the impact of feeding diets rich in polar lipids on the plasma lipidome of piglets during the weaning period. **Material and Methods:** Weaned male piglets (*n* = 240; 21 days of age; 6.3 ± 0.5 kg of BW) were blocked by initial weight and distributed into 48 pens of five animals each in a complete randomized block design with a 2 × 3 factorial arrangement of treatments as follows: a plant-based diet rich in neutral lipids from soybeans (24 pens; SD) or a polar lipid-rich diet by-product of cheese making (24 pens; PD) from weaning until the 21st day of the nursery phase. Within each diet group, animals received one of three milk replacers (MR; 0.5 L/d/animal) for the first 7 days after weaning: (1) commercial MR containing animal and coconut lipids (CO); (2) polar lipid-based MR (PO); or (3) soybean lipids-based MR (SO). **Results:** The PD diet group increased the plasma concentrations of sphingolipids, phospholipids, and cholesterol esters, but did not impact the concentrations of glycerolipids (GLs). Both the PO and CO milk replacers increased the plasma concentrations of ceramide, acyl-chain phosphatidyl choline, and cholesterol esters. The plasma concentrations of GLs containing 18-carbon fatty acids such as 18:0, 18:1, 18:2, and 18:3, were higher in SD, whereas GLs containing 16:0 and 20:3 were higher in PD. **Conclusions:** In summary, the diet lipid type significantly modulated the plasma lipid composition in piglets 7 days after weaning. The dietary inclusion of polar lipids in diets for growing pigs can modulate the plasma lipidomic profile, relative to plant-based diets rich in soybean lipids. Cost may be a major consideration when using these lipids in pig diets. Their health benefits need to be further characterized in other models of stress and inflammation.

## 1. Introduction

The abrupt transition from a liquid to a solid diet during the weaning period leads to a reduction in feed intake and changes in the intestinal structure in piglets [1]. Villous atrophy and crypt elongation impair the function of the small intestine, increasing permeability and triggering inflammatory processes [2]. Potentiated by psychosocial and immunological stressors, inflammation compromises the health and performance of animals [3].

Diet, as a modulator of different aspects of intestinal health, can enhance or minimize these inflammatory effects on the basis of its composition [4]. Vegetable protein sources such as soy can be used in piglet diets, but the potential for digestive issues limits their inclusion. However, in addition to being challenging to the immature intestine of young piglets, these dietary ingredients contain antinutritional factors and proinflammatory n-6 lipids, such as linoleic acid (LA; 18:2 *cis*-9, *cis*-12; [3]). Although soybeans contain lipids with anti-inflammatory functions (e.g., sphingolipids), their concentrations are typically low (1.6–2.0% phospholipids in whole beans; [5]) compared to polyunsaturated fatty acid (PUFA)-rich triglycerides (TGs), which represent around 64% of total lipids [6]. Furthermore, the high proportions of n-6 PUFA present in grains and vegetable oils can increase oxidative stress and the production of proinflammatory mediators, mainly through the arachidonic acid pathway [7]. Pigs fed a diet with high n-6/n-3 ratios showed lower energy digestibility and weight gain rates [8], which could have been associated to reduced inflammation and changes in metabolism [9].

On the other hand, polar lipids from the globular membranes of bovine milk fat, such as glycerophospholipids and sphingolipids, are lipid sources that can induce anti-inflammatory and protective effects on intestinal integrity [10]. Sphingomyelin is present in approximately 75% of bovine milk sphingolipids [11]. Young mice fed a lard-based diet containing 0.1% by weight of sphingomyelin of bovine dairy origin for 10 weeks had reduced hepatic accumulation of triglycerides and circulating inflammatory cytokines, attenuating the inflammatory responses of macrophages after pro-inflammatory stimuli [10]. Furthermore, the supplementation of polar lipids from milk fat globular membranes during gestation resulted in reduced gut permeability and the improved growth of neonatal pigs [12]. Sphingomyelin exhibits antimicrobial, antioxidant, and neuroprotective effects [13], and it can be a substantial dietary source of phosphocholine, and, thus, promote neonatal development [14]. Sphingosine-1-phosphate (S1P) is the phosphorylated product of sphingosine, the backbone of sphingomyelin and other sphingolipids. Importantly, S1P is considered a regulator of inflammation given its roles in reducing T-cell proliferation and cytokine synthesis in the intestinal tract [15].

Lipidomics is a powerful tool for investigating the modulation of metabolism during normal and disease states. Understanding the response of cellular metabolism to a dietary intervention by quantifying changes in diverse lipid classes and species can be associated to changes in gene expression and enzyme activity [16], which can improve our understanding of the potential impact of diet on performance and animal health. Since complex dietary lipids such as sphingolipids are not absorbed intact in the intestine [17], the plasma concentration of hydrolyzed lipids could serve as a proxy for their digestion and metabolism.

We hypothesized that the polar lipids in the diet and milk replacers would result in extensive changes in the plasma lipidomic profile of growing piglets, which can be associated with modulation of inflammatory markers during the first 7 days after weaning. The objective was to evaluate the impact of a modified by-product of cheese-making rich in polar lipids on the plasma lipidomic profiles of piglets during the weaning period. In addition, we aimed to study the influence of the dietary fatty acid profile on plasma lipid composition.

## 2. Materials and Methods

### 2.1. Experimental Design and Treatments

All procedures were approved by the animal care committee (2022-PO-440) of the Centre de Recherche en Sciences Animales de Deschambault (CRSAD), in Deschambault, Canada, following the regulations of the Canadian Council on Animal Care (1993).

**Figure 1:**
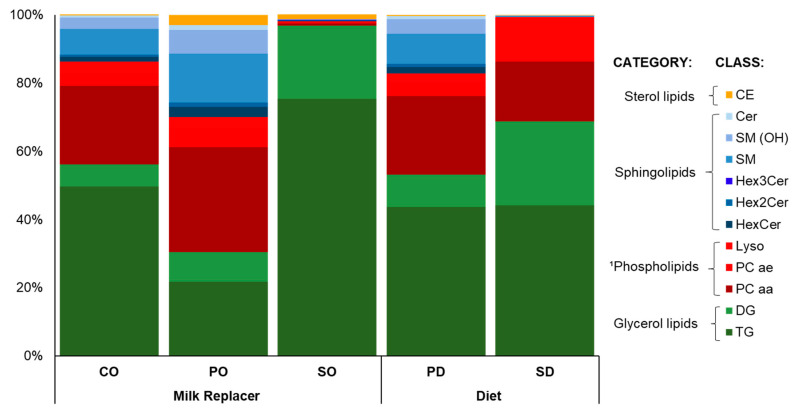
Overall concentration of diverse lipid classes identified in experimental milk replacers and diets. Treatments were (1) milk replacer, commercial milk substitute rich in animal fat and coconut oil (CO), milk substitute rich in polar lipids, (PO) or milk substitute rich in soy lipids (SO); and (2) diet, solid feed containing soy lipids (SD) or lipids from cow milk fat globular membranes (PD). Sterol lipids: cholesteryl ester (CE). Sphingolipids: ceramide (Cer); sphingomyelin (SM); sphingomyelin with a hydroxyl group (SM (OH)); ceramide with a hexose sugar residue attached to the sphingoid base and a fatty acid chain (HexCer); ceramide with two hexose sugar residues attached to the sphingoid base and a fatty acid chain (Hex2Cer); and ceramide with three hexose sugar residues attached to the sphingoid base and a fatty acid chain (Hex3Cer). ^1^Phospholipids containing choline: phosphatidylcholine with an acyl chain (PC aa); phosphatidylcholine with an acyl-alkyl chain (PC ae); and lysophosphatidylcholine (Lyso). Glycerolipids: diacylglyceride (DG) and triglyceride (TG). The details in lipid composition of treatments as % of total lipids are shown in Supplemental Table S1.

A total of 240 weaned male piglets (25% Landrace, 25% Yorkshire and 50% Duroc) of 21 days of age were used in this study. Animals were blocked by initial weight (6.3 ± 0.5 kg) and distributed into 48 pens of five animals in a complete randomized block design with a 2 × 3 factorial treatment arrangement. From the start of the nursery phase, animals received one of two diets ad libitum: (1) soy diet (SD), a feed containing soy lipids where sphingomyelin (SM), phospholipids containing choline (PC), and triglyceride (TG) were 0.40, 18, and 44% of total lipids, respectively (24 pens); or (2) polar diet (PD), a diet containing polar lipids from a phospholipid- and protein-rich by-product of cheese making (Iso-Chill 6000, Agropur, Dairy Cooperative. St-Hubert, QC, Canada), where SM, PC, and TG were 13, 27, and 44% of total lipids, respectively (24 pens). From the start of the nursery phase to day 7, animals within each dietary group were offered one of three milk replacers (0.5 L/pig/day; consumed completely in all groups): control milk substitute (CO), a commercial product composed of animal fat lipids and coconut oil (SM, PC, and TG were 11, 27, and 50% of total lipids, respectively); (2) milk substitute rich in polar lipids (PO), containing a high-fat whey protein concentrate (Isochill 6000, Agropur, QC, Canada; SM, PC, and TG were 21, 36, and 22% of total lipids, respectively); or (3) milk substitute composed of vegetable lipids (SO), containing lipids from soybeans (SM, PC, and TG were 0.2, 0.8, and 75% of total lipids, respectively). Lipid concentrations, including sphingolipids, glycerolipids, phospholipids, and sterol lipids, are shown Figure 1 and Supplemental Table S1. All piglets received vaccines for Porcine Circovirus type 2 (Ingelvac Circoflex; Boehringer Ingelheim Ltd., Burlington, ON, Canada) and *Mycoplasma Hyopneumoniae* (Ingelvac Mycoflex; Boehringer Ingelheim Ltd., Burlington, ON, Canada). A total mortality rate of 1.6% was observed during the experiment, corresponding to one animal in the CO group, one in the PO group, and two in the SO group.

The feed was formulated based on NRC (2012) requirements for digestible amino acids and metabolizable energy. Diets were formulated to be iso-energetic and iso-proteic. Feed samples were collected once a week and stored at −20 °C until further analyzed.

Blood samples were collected on day 7 from three randomly selected piglets in every pen. Samples were kept on ice and centrifuged within 30 min at 4 °C 3000× *g* to obtain plasma, which was then pooled by pen (*n* = 8 pooled samples per treatment) and conserved at −80 °C until analyzed for the concentrations of lipopolysaccharide-binding protein (LBP) by immunoassay (MyBiosource; Swine LBP ELISA kit, San Diego, CA, USA) and for lipidomic analyses as described below. Fecal samples from three animals per pen were collected on day 7 after placing piglets in individual crates where they were allowed to defecate. These samples were pooled by pen, snap-frozen, and stored at −80 °C until analyzed for calprotectin concentrations by ELISA (MyBiosource; Swine Calprotectin kit).

### 2.2. Feed Composition Analyses

Diets and milk replacers were sampled once per week and pooled by treatment for the experimental period, then analyzed for humidity, crude protein, crude fiber, and minerals by wet chemistry (SGS Canada Inc., Guelph, ON, Canada; Table 1). Fatty acids (FA) were directly trans-esterified [18] and methyl esters were identified and quantified in a gas chromatograph (Agilent 7820A; Agilent Technologies Canada Inc. Mississauga, ON, Canada) equipped with a HP-INNOWax column (30-m length × 0.32-mm internal diameter × 0.25-μm film thickness; Agilent Technologies Canada Inc.) and a flame ionization detector with hydrogen as the carrier gas [19]. Total fatty acid content of feeds was determined using 13:0 and 21:0 as internal standards (Sigma Aldrich, Diegem, Belgium). Dietary fatty acid profiles are shown in Table 2.

### 2.3. Feed and Plasma Lipidomic Analysis

A lipidomic analysis was performed on feed and plasma samples for determination of cholesteryl ester (CE), ceramide (Cer), sphingomyelin (SM), sphingomyelin with a hydroxyl group (SM (OH)), ceramide with a hexose sugar residue attached to the sphingoid base and fatty acid chains (HexCer), ceramide with two hexose sugar residues attached to the sphingoid base and fatty acid chains (Hex2Cer), ceramide with three hexose sugar residues attached to the sphingoid base and fatty acid chains (Hex3Cer), phosphatidylcholine with an acyl chain (PC aa), phosphatidylcholine with an acyl-alkyl chain (PC ae), lysophosphatidylcholine (Lyso), diacylglyceride (DG), and triglyceride (TG) concentrations (The Metabolomics Innovation Center; AB, Canada). Feed samples were homogenized with three-fold volume of extraction buffer consisting of 85 mL of MeOH + 15 mL of phosphate buffer (10 mM). Samples were then centrifuged at 14,000 rpm and supernatants used in the lipidomics assay were as described for plasma below.

Plasma samples were thawed on ice in the dark before use. In a 96-well filter plate, 20 μL of the internal standard mixture solution [including 9:0 Lyso PC, 6:0 SM, 14:0 PC, Cer(d18:0/12:0(OH)), CE(16:0)-d7, DG(17:0_17:0)-d5, GlcCer(d18:1/12:0), and TG(16:0_34:0)-d5 as internal standards; and 18:0 Lyso PC, 18:0 SM, 36:0 PC, Cer(d18:1/18:0), CE(17:0), DG(18:1/18:1), LacCer(d18:1/18:0), GlcCer(d18:1/18:0), and TG(18:1/18:1/18:1)] and 10 μL of sample was pipetted directly onto the center of the spot. The plate was evaporated for 30 min to dryness under nitrogen, followed by the addition of 300 μL of methanol containing 5 mM ammonium acetate. The plate was covered and shaken at 450 rpm for 30 min at room temperature, and then centrifuged for 5 min at 500 rpm. After centrifugation, 10 μL of the extracts was transferred to a new 96-well collection plate and mixed thoroughly with 490 μL of running solvent (60 μL of formic acid and 10 mL of water in 290 mL of methanol). Then, 20 μL was injected into an Agilent 1290 series Ultra high-performance liquid chromatography (HPLC) system (Agilent Technologies, Palo Alto, CA, USA) equipped with a tandem mass spectrometry instrument (ABSciex 5500 QTrap^®^, Applied Biosystems/MDS Analytical Technologies, Foster City, CA, USA) equipped with an Ultra high performance liquid chromatography (HPLC) system (Agilent Technologies, Palo Alto, CA, USA). Data analysis was performed using MultiQuant TM 3.0.3 (Applied Biosystems/MDS Analytical Technologies, Foster City, CA, USA). The UHPLC autosampler was connected directly to the MS ion source by red PEEK tubing. The running buffer was used as the mobile phase, while the flow rate was programmed as follows: t = 0 to 1.6 min, 30 μL/min; t = 2.4 to 2.8 min; 200 μL/min; and t = 3.0 min, 30 μL/min. The sample injection volume was 20 μL. The IonSpray voltage was set at 5500 V and the temperature was set at 200 °C. The curtain gas, gas pressure 1, gas pressure 2, and collision-induced dissociation were set at 20, 40, 50, and medium, respectively. The entrance potential and collision cell exit potential were set at 10 and 15 V, respectively.

### 2.4. Statistical Analysis

Data were analyzed using the web-based platform MetaboAnalyst 5.0 [20]. Non-filtered data were normalized by the sum method, generalized log-transformed, and Pareto-scaled. Multivariate analysis of data included partial least squares discriminant analysis (PLS-DA), ANOVA, and Pearson’s correlation coefficient procedures. Significance was declared at a false discovery rate (FDR) < 0.05. For visualization purposes, heat maps were generated to showcase the magnitude of fold-change in a color gradient for increased (red) or decreased (blue) relative abundance.

Data found to be significantly affected by treatments were then analyzed in a mixed model in SAS 9.4 (The SAS Institute Inc., Cary, NC, USA) including pen as a random effect, and milk replacer, a diet, and their interactions were parametrized as fixed effects. Significant differences were considered when *p* ≤ 0.05 for main effects and *p* ≤ 0.10 for interactions; trends for differences were considered when *p* ≤ 0.10 for main effects and *p* ≤ 0.15 for interactions.

## 3. Results

The reversed-phase LC-MS/MS assay identified 404 lipids in the diet (Figure 1; Supplemental Tables S1 and S2) and in the plasma of piglets (Supplemental Table S3) that can be grouped into 12 classes belonging to four lipid types: sphingolipids, sterol lipids, a subgroup of phospholipids (i.e., those containing choline), and glycerolipids. The sphingolipids detected included 9 SM, 5 SM (OH), 18 Cer, 17 HexCer, 7 Hex2Cer, and 4 Hex3Cer. The phospholipids subgroup included 14 Lyso, 37 PC aa, and 38 PC ae. The glycerolipids included 21 DG and 212 TG. Finally, sterol lipids were represented by 22 CE.

In solid feeds, the lipid class concentrations varied according to the composition of each dietary treatment (Figure 1; Supplemental Table S1). Relative to SD, the PD diet contained higher concentrations of sphingolipids (i.e., 31-fold) and cholesterol esters (i.e., five-fold), equal concentrations of phospholipids, and lower concentrations of glycerolipids (i.e., −23%). Similarly, relative to CO and SO, the PD milk replacer contained higher concentrations of sphingolipids (i.e., two- and 40-fold, respectively), cholesterol esters (i.e., 11- and two-fold, respectively), and phospholipids (1.3- and 31-fold), and lower concentrations of glycerolipids (i.e., −46% and −69%).

The fatty acid (FA) profiles of the treatments showed higher concentrations of 12:0, 14:0, and 16:0 in polar lipid-rich treatments (PO and PD; Table 2). Meanwhile, CO and SO milk replacers and the SD diet presented a higher concentration of 18:1. In addition, the SD diet group exhibited higher concentrations of 18:2n6 and 18:3n3 compared with PD.

### Effects of Milk Replacer and Diet on Plasma Lipids of Weaned Piglets

Two-factor analysis revealed no interaction between milk replacer and diet for plasma lipids (*FDR* > 0.05). The factors were then analyzed individually.

**Figure 2:**
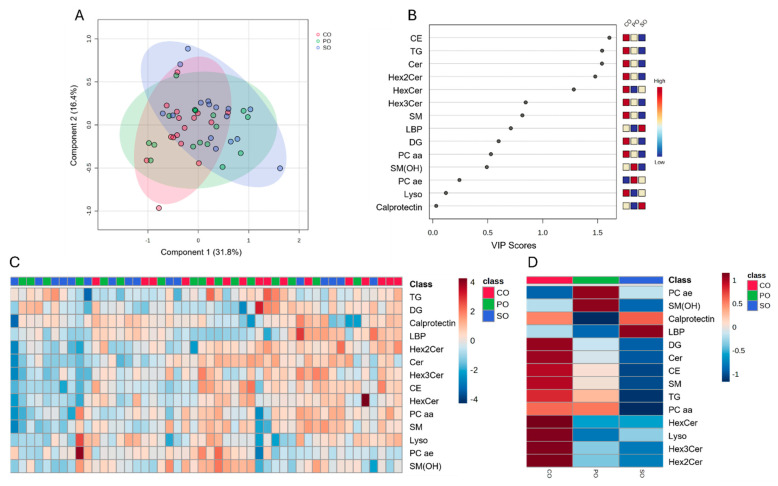
Discriminant analysis and hierarchical grouping of plasma lipid classes measured on day 7 of the nursery phase in piglets fed commercial milk substitute rich in animal fat lipids and coconut oil (CO), milk substitute rich in polar lipids (PO), or milk substitute rich in soy lipids (SO). (**A**): two-dimensional partial least squares discriminant (PLS-DA) score plot. (**B**): variable importance projection (VIP) scores analysis based on component 1 of the PLS-DA used to rank the relative contribution of lipids to the variance between treatments. (**C**): heatmap clustering analysis of the plasma lipid classes influenced by milk replacer treatments. (**D**): heatmap group averages. Plasma lipids data were obtained using a LC–MS/MS custom assay.

Partial least squares discriminant analysis (PLS-DA) plots of the plasma lipid data revealed no clear clustering by MR group (i.e., CO, PO and SO) (Figure 2A). However, the variable importance projection (VIP) scores analysis showed that CE, TG, Cer, Hex2Cer, and HexCer had VIP scores > 1 (Figure 2B). These lipids were highest in CO, medium in PO, and lowest in the SO milk replacer treatment. The heatmap revealed no clear clustering of lipid type concentrations by milk replacer (Figure 2C). The heatmap clustering (Figure 2D) shows higher concentrations of calprotectin and LBP in the SO group compared to other MR, although mixed-model analysis revealed no significant differences.

**Figure 3:**
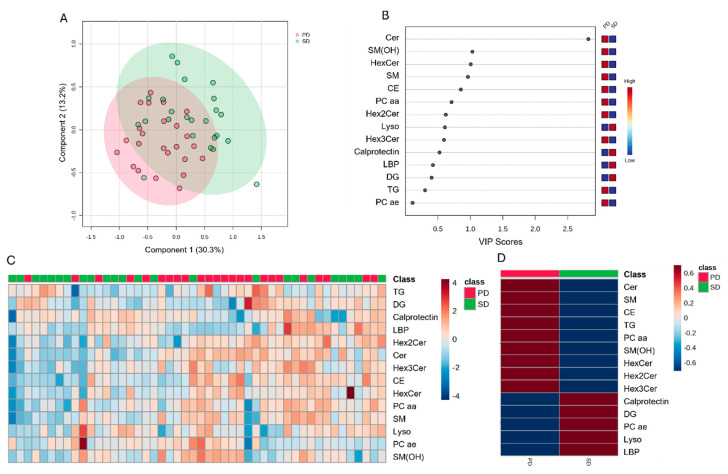
Discriminant analysis and hierarchical grouping of plasma lipid classes measured on day 7 of the nursery phase in piglets fed a diet rich in soy lipids (SD) or a diet rich in polar lipids from cow’s milk fat globular membranes (PD). (**A**): two-dimensional partial least squares discriminant (PLS-DA) score plot. (**B**): variable importance projection (VIP) scores analysis based on component 1 of the PLS-DA used to rank the relative contribution of lipids to the variance between treatments. (**C**): heatmap clustering analysis of the plasma lipid classes influenced by treatments. (**D**): heatmap group averages. Plasma lipids data were obtained using a LC–MS/MS custom assay.

**Figure 4:**
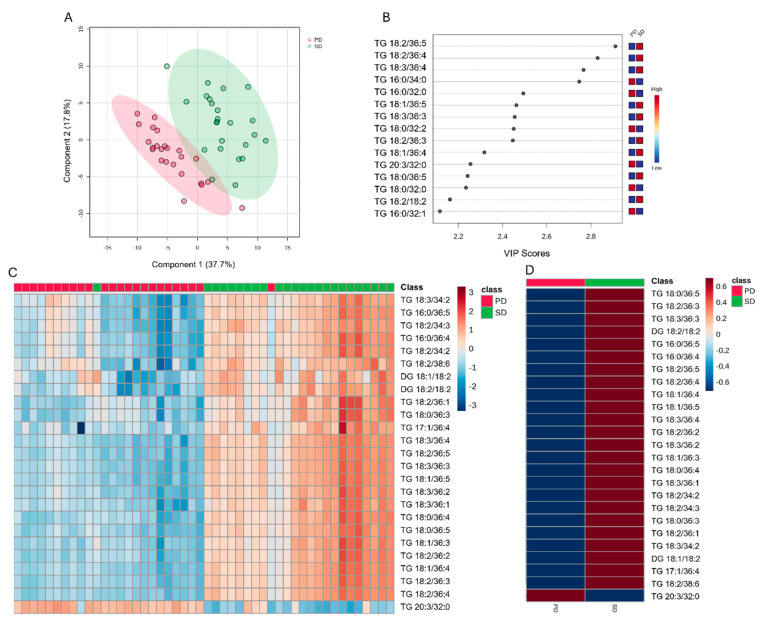
Discriminant analysis and hierarchical grouping of plasma lipid concentrations on day 7 of the nursery phase in piglets fed a diet rich in soy lipids (SD) or a diet rich in polar lipids from cow milk fat globular membranes (PD). (**A**): two-dimensional partial least squares discriminant (PLS-DA) score plot. (**B**): variable importance projection (VIP) scores analysis based on component 1 of the PLS-DA used to rank the relative contribution of lipids to the variance between treatments. (**C**): heatmap clustering analysis of the plasma lipid classes influenced by milk replacer treatments. (**D**): heatmap group averages. Plasma lipids data were obtained using a LC–MS/MS custom assay.

Similarly, the PLS-DA plots of the plasma lipid data revealed unique features predictive of SD and PD diets (Figure 3A). The VIP scores analysis showed that only Cer had a VIP score > 1 (Figure 3B), being the highest in the PD treatment. Heat maps showed no clear clustering of lipid type concentrations by diet (Figure 3C). The heatmap analysis (Figure 3D) also showed higher concentrations of calprotectin and LBP in the SD compared with the PD group. However, when the 404 lipids identified in piglets’ plasma were analyzed individually, the discriminant analysis revealed a clear clustering by diet type (PD and SD; Figure 4A). The VIP scores analysis showed the top 15 lipids that had a VIP score > 1, namely, TG containing 16:0, 18:0, 18:1, 18:2, 18:3, and 20:3 fatty acids (Figure 3B). The heatmap analysis of the 25 top clustered lipids (Figure 4C,D) showed that TG containing fatty acids such as 16:0, 17:1, 18:0, 18:1, 18:2, and 18:3 were elevated in the SD group, as were DG containing 18:1/18:2 and 18:2/18:2 fatty acids. On the other hand, the PD group exhibited a higher concentration of TG containing 20:3 carbon fatty acids.

The mixed-effects model analysis revealed an effect of both diet and milk replacers on the plasma concentrations of CE, Cer, HexCer, Hex2Cer, Hex3Cer, PC aa, and SM (OH; *p* < 0.05; Table 3), whereas glycerolipids remained unchanged. More specifically, compared with the SD diet group, PD resulted in higher plasma concentrations of SL, PC aa, and CE (*p* < 0.05). In addition, relative to SO, both the CO and PO milk replacer groups exhibited higher plasma concentrations of Cer, PC aa, and CE (*p* < 0.05), whereas SM(OH) concentrations were higher in PO compared to CO and SO (*p* < 0.05).

## 4. Discussion

The use of milk replacers during the weaning period is not a common practice in pig farming due to the increase in production costs. However, the use of milk replacer can reduce the oxidative stress associated with feeding adaptation and intestinal maturation that piglets face during weaning in intensive farming systems [10]. Our study investigated the impact of different types of milk replacers in the first seven days after weaning, as this is the most critical period of this adaptive phase. Indeed, given the drastic change in diet (i.e., liquid to solid), feed intake and growth are depressed, and animals can develop leaky gut, inflammation, and oxidative stress [21]. Although it is not a common practice to provide milk replacers to all animals during the nursery phase (i.e., after weaning), our interest was to offer a liquid source of dietary lipids to counter the low solid feed intake observed during the first week, and, thus, to maximize the ingestion of these lipids during this period.

We tested a source of polar lipids from milk fat globular membranes obtained as a by-product of cheese-making and a soy source rich in PUFA, since different dietary lipid precursors, when hydrolyzed and absorbed by the intestine, can influence tissue metabolism and inflammatory biomarkers in different ways [22]. Furthermore, an important aspect of considering the use of polar lipids as a potential feed ingredient in diet for piglets was the potential to be easily obtained from dairy-processing factories as a by-product of cheese making, which may make it a viable alternative to other sources of lipids and proteins depending on the market price. Importantly, the future use of these products in swine feeding will also require an evaluation of optimal inclusion levels and effects on performance.

We measured the plasma concentrations of diverse lipid classes on day 7 of the experiment as the cumulative effect of diet and milk replacer was expected to be highest at this time. Our lipidomic analysis allowed to identify and quantify 404 different lipid types in the MR and diet treatments, which were grouped into the 12 classes. Globally, the predominant lipid type across treatments was glycerolipids (TG and DG), followed by sphingolipids (e.g., SM) and phospholipids (PC aa); however, the proportions varied greatly by treatment. Relative to milk fat globule membrane (MFGM) lipids, whole milk fat contains significantly higher concentrations of TG (i.e., 98% of total lipids), whereas the remainder is composed mainly of polar lipids, which, themselves, can vary significantly in composition [11,23]. Brink et al. [24] evaluated the variability in the composition of commercially available MFGM products for infants and found 338 lipid species. In their study, among the lipid classes with the highest relative abundance in a product derived from whey, TG was the highest, around 70%, followed by PC with 20% and SM 10%. In contrast, the PD diet contained 22, 31, and 14% of TG, PC, and SM, respectively, while the PO milk replacer contained 44, 23, and 9% of TG, PC, and SM, respectively. Such variation between available products may be the result of different manufacturing practices and substrates, which may be an important consideration since it may influence the impact of these products on animal metabolic and growth responses.

Our results did not demonstrate an interaction between the MR and diet treatments, but there were significant changes in plasma lipids when analyzed as individual factors. The descriptive analysis of the lipids grouped into 12 classes for MR and diet did not show clear clustering; however, when the 404 lipids identified in the piglet plasma were analyzed individually, a clear clustering (i.e., metabolite grouping) by basal diet type was revealed, which indicates that dietary modifications are able to alter individual lipid species, perhaps reflecting changes in metabolism. Furthermore, the reason for treatment clustering only based on diet and not on the milk replacer type is not clear. Based on the amount of MR offered (0.5 L/animal/d), approximately 80 g of lipids from the MR were ingested. Although solid feed intake was not measured on day 7, typical intakes vary between 100 and 200 g/d, which would provide 6–12 g/d of lipids from the feed during this period. Therefore, diet clustering may be explained by other factors.

Among the 25 lipids with the highest plasma concentrations in SD, 23 were TG, mainly from species 18:0, 18:1, 18:2, and 18:3, and two were DG 18:1 and 18:2. Furthermore, the plasma concentration of glycerolipids (TG and DG) represented 68.7% of the total lipids identified in the SD treatment group. These results suggest a dietary origin, that is, a soy-based diet that predominantly has 18-carbon lipids in its composition, varying around 70% of the total FA depending on the soy source used [6]. Corroborating this, the dietary fatty acid profile demonstrated two and four times more 18:2n6 and 18:3n3, respectively, in the SD compared to the PD treatment. Combined with a lower concentration of 18:0 in the fatty acid profile of the SD group, it is suggested that the concentrations of 18:3, 18:2, and 18:1 present in plasma were of dietary origin, which presented a high plasma concentration in the SD plasma.

When comparing the FA profiles of the treatments, FA 18:0 presented a concentration twice as high in the PD diet profile compared to SD, and 18:1 presented similar concentrations in all treatments. Sphingosine with an 18-carbon chain and a cis double bond (d18:1) is the most abundant sphingoid base in mammalian cells [17], which explains the high concentrations of 18:1 in the fatty acid profile of diets of animal origin, PD, PO, and CO. Although 18:1 was equal in concentration to plant-based treatments, the fatty acid with the highest proportion in the lipid profile of polar lipids treatments (PD and PO) was 16:0. In addition, dietary 12:0 and 14:0 concentrations were higher in PO and PD, probably due the presence of coconut oil in these treatments [25]. Although medium-chain fatty acids are absorbed intact from the intestine [26], the top group-discriminant plasma lipids (TG and DG) did not contain these fatty acids.

**Figure 5:**
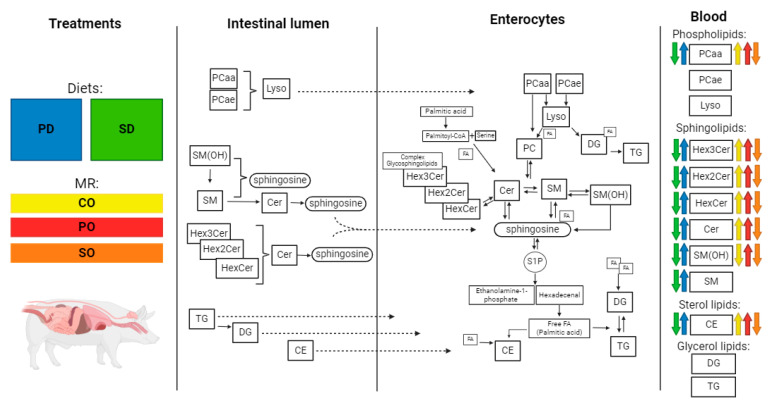
Digestion and absorption pathways of lipid classes identified in the diet and in milk replacer (MR) treatments. The treatments were (1) milk replacers, commercial milk substitute rich in animal fat lipids and coconut oil (CO), milk substitute rich in polar lipids (PO), or milk substitute rich in soy lipids (SO); and (2) diets, solid feed containing soy lipids (SD) or polar lipids from cow’s milk fat globular membranes (PD). The intestinal lumen presents the classes of lipids identified from the analysis of dietary treatments and their possible metabolization before being absorbed into the enterocytes. Phosphatidylcholine with an acyl chain (PCaa) and phosphatidylcholine with an acyl-alkyl chain (PCae) are precursors of lysophosphatidylconine (Lyso) and can be transformed into Lyso in the intestinal lumen. Sphingomyelin with a hydroxyl group (SM(OH)) can be transformed into sphingomyelin (SM) or directly into sphingosine. SM is not absorbed into the intact enterocyte and is broken down into ceramide (Cer) in the intestinal lumen. Similarly, complex lipids such as ceramide with a hexose sugar residue attached to the sphingoid base and fatty acid chains (HexCer), ceramide with two hexose sugar residues attached to the sphingoid base and fatty acid chains (Hex2Cer), and ceramide with three hexose sugar residues attached to the sphingoid base and fatty acid chains (Hex3Cer) are not absorbed intact and are broken down into Cer. Cer is also hydrolyzed to sphingosine and fatty acids (FA). Sphingosine is then absorbed by enterocytes. Dietary triglycerides (TG) can be absorbed intact or broken down into diacylglycerides (DG) and free FA. Cholesterol ester (CE) is absorbed intact by the enterocyte. In the enterocyte, PCaa can produce Lyso or directly phosphatidylcholine (PC), which, together with Cer, constitutes SM. SM can acquire a hydroxyl group forming SM(OH) and be transformed back into Cer and PC, or it can be hydrolyzed to sphingosine. Cer can be transformed into SM when added to a PC, be hydrolyzed into sphingosine, or form complex sphingolipids via the salvage pathway. Sphingosine can be absorbed from the intestinal lumen or be the product of de novo synthesis from palmitic acid and serine. A small amount of sphingosine goes into the blood circulation and tissues to be integrated into SM and Cer or is catalyzed to generate sphingosine-1-phosphate (S1P). S1P is degraded to ethanolamine and hexadecenal phosphate. Hexadecenal is converted into palmitic acid, which can be esterified into DG and TG or transformed in CE. Blood has all identified lipid classes and the colored arrows represent an increase or decrease in their concentrations in the respective dietary treatments; green represents SD; blue represents PD; and yellow, red, and orange represent the CO, PO, and SO milk replacers. Lipid classes without colored arrows did not differ between treatments. Figure created in BioRender.com.

The concentration of 16:0 was 1.9 times higher in PD, relative to the SD diet group, which was also reflected in a high transfer of this fatty acid to the plasma of piglets in PD (Figure 4B). Sphingolipids such as SM and Cer are not absorbed intact from the intestine; for example, SM is hydrolyzed by Alk-SMase to Cer and to sphingosine and free fatty acids (FA) by N-CDase in the intestinal lumen (Figure 5) [10]. Sphingosine, the backbone of most sphingomyelins, is absorbed well by the enterocyte. It is primarily phosphorylated to generate sphingosine-1-phosphate by sphingosine kinase (SoK; [27]). The remaining sphingosine is transported by chylomicrons to the lymph and blood. Sphingosine-1-phosphate can be dephosphorylated by S1P phosphatase (S1PP) to a new sphingosine to form Cer or degraded by S1P lyase to ethanolamine phosphate and hexadecenal [28]. Hexadecenal, in turn, is converted into free FA and can be, then, esterified into TG or CE (Figure 5). Furthermore, 16:0 can be activated by conversion to palmitoyl-CoA, and in turn, used to promote de novo ceramide synthesis by its condensation with serine residues [29]. While several molecular species may be synthesized, only a handful of them have been linked to disorders of metabolism. For example, C16:0-Cer is a pro-inflammatory molecule and it appears to play a role in the pathogenesis of obesity and diabetes [30]. Indeed, the fatty acid composition of different TG species will be dependent on diet lipid composition and will be associated to its potential to exert pro- or anti-inflammatory effects [31]. The identity of other FA in 16:0-containing TG is, therefore, expected to influence its overall effect on metabolism.

TG concentrations containing 20:3 were higher in the plasma of PD piglets. However, the FA profile of dietary feeds did not identify 20-carbon fatty acids with three unsaturations, which may be due to coelution with other long-chain fatty acids or metabolism into other FA. A small fraction of 20:3 can be desaturated to 20:4, while the main amount is normally metabolized via the cyclooxygenase pathway into series 1 prostaglandins, having anti-inflammatory actions [32].

Although it was the class of sphingolipids with the highest plasma concentration, SM was the only one that showed an effect from diet only, suggesting alterations in lipid metabolism in MR treatments. As mentioned, dietary SM is not absorbed intact, that is, the amount found in plasma is endogenously reconstituted SM. SM is formed by Cer and a phosphocholine (PC) originating from the hydrolysis of lysophosphatidylcholine (Lyso) whose precursors are phosphatidylcholines (PCaa and PC ae; Figure 5). CE, phospholipids (PL), and TG represent 36, 30, and 16% of total lipids in plasma, respectively [33]. In swine plasma, phosphatidylcholine is the PL with the highest concentration, followed by SM and Lyso [29], which corroborates our findings (Table 2). Furthermore, PCaa was shown to be the main source of PC in the production of SM. Even though PCaa had the highest concentration among dietary phospholipids (Figure 1), this was the only lipid class in the phosphocholine category that was increased by the PD diet and also by the CO and PO milk replacers in MR.

Sphingomyelin, in addition to being reverted to Cer, can acquire a hydroxyl group (SM(OH))—which is also a direct source of sphingosine—and/or be hydrolyzed into sphingosine and free fatty acids (Figure 5) [17]. Hydroxylated SM, although less concentrated in plasma than SM, was regulated by diet and MR, being greater in the treatments containing polar lipids (PD and PO). Since SM was unaffected, but SM(OH), Cer, and PCaa were altered in the same treatment groups, this suggests that SM was metabolized through these pathways.

Plasma Cer, although found in lower concentrations compared with SM, was modulated by both diet and the MR treatments, being higher in the treatments with polar lipids (PD and PO) and CO. This class can be hydrolyzed by SoK to sphingosine and free FA, as previously described, but can also be a precursor of SM and complex lipids [17]. To the formation of complex sphingolipids through the salvage pathway, sugars are gradually added to their chain, forming HexCer, Hex2Cer, and Hex3Cer, respectively (Figure 5). This corroborates the gradual concentrations and differences between treatments for these classes, showing a Cer conversion.

Although not measured, an increase in S1P concentration is suggested due to the plasma increase in its precursors (i.e., SM present in animals fed polar lipid-rich feeds). S1P is a bioactive molecule with immunosuppressive and anti-inflammatory properties. It was expected that with the supply of SM in the diet, sphingosine phosphorylations would increase in the inflamed enterocytes of weaned piglets and reduce the proliferation of T cells and the synthesis of cytokines, controlling the inflammatory process and all its negative effects on metabolism [15]. Although our data on inflammatory markers did not show significant differences between treatments, it is likely that no underlying inflammatory processes were at play. Indeed, in contrast to commonly used models of weaning stress [21,34,35], ours included access to MR in all groups during the first week; animals had the opportunity to progressively adapt to the solid feed without relying exclusively on it from the start of the nursery phase. Although the plant-based lipids such as linoleic acid can act as precursors of pro-inflammatory oxylipids [36], the fact that the piglets ingested a milk replacer may have been enough to reduce the degrees of stress and inflammation typically observed during weaning.

## 5. Conclusions

Our results demonstrate large individual variation in plasma lipids according to dietary lipid composition in piglets 7 days after weaning. The lipid profiles of dietary treatments, in particular from the solid portion of the diet, altered the plasma lipid composition, resulting in increased circulating sphingolipids, phospholipids (i.e., PCaa), and cholesterol esters in the groups receiving polar lipid supplementation. Interestingly, changes in individual lipids were predominantly observed for TG and DG species, which corresponded with the dietary fatty acid profiles.

The inclusion of polar lipids in diets for growing pigs can modulate the plasma lipidomic profile relative to plant-based diets rich in soybean lipids. Future research is necessary to evaluate the extent of the impact of such changes in the plasma lipidomic profile on overall metabolism, inflammation, and growth performance. In addition, the future use of polar lipids in the swine industry will depend on their availability as feeds and their market price in comparison to other sources of fat and protein.

## Figures and Tables

**Table 1:** Ingredient and chemical compositions of experimental milk replacers (MR) and diets.

Ingredients, %	MR			Diet	
	CO	PO	SO	PD	SD
Lactoserum powder	35.9	51.2	46.6	-	-
Ground corn	-	-	-	21.9	28.9
WPC 50% ^1^	-	31.5	-	20.0	-
Ground wheat	-	-	-	18.2	-
Wheat flour	-	-	-	-	14.6
Corn gluten meal	-	-	-	15.0	14.6
Ground beet pulp	-	-	-	10.0	-
Soy protein concentrate	-	-	19.1	-	11.6
Ground soybean meal 48% CP	-	-	-	-	9.75
Animal fat	23.8	9.5	11.0	-	-
Coconut fat	0.9	3.8	-	-	-
WPC 80% ^2^	21.4	-	11.0	-	-
WPC 34% ^3^	15.1	-	-	-	-
Soybean oil ^4^	-	-	9.2	-	-
Dicalcium de phosphate	1.2	-	1.3	-	-
L-Lysine	-	1.0	-	-	-
Others	1.7	3.0	1.8	4.83	8.59
Chemical composition, %				-	-
Humidity	4.5	4.5	4.9	9.49	9.45
Crude Protein	28.0	27.6	26.6	19.4	21.2
Fatty acids	15.3	15.4	16.4	6.32	7.65
Crude fiber	<0.1	0.2	0.2	4.29	3.07
Metabolizable energy (Mcal/kg)	4.0	4.1	4.2	3.39	3.52
Ash	6.7	6.0	6.8	5.37	5.63
Calcium	0.5	0.6	0.6	0.71	0.61
Phosphorus	0.6	0.6	0.7	0.60	0.72
Potassium	1.0	1.2	1.4	0.40	0.84
Magnesium	0.03	0.04	0.1	0.12	0.13
Sodium	0.8	0.7	0.7	0.25	0.39

Treatments were (1) milk replacer, commercial milk substitute rich in animal fat and coconut oil (CO), milk substitute rich in polar lipids (PO), or milk substitute rich in soy lipids (SO); and (2) diet, solid feed containing soy lipids (SD) or lipids from cow milk fat globular membranes (PD). ^1^ Whey protein concentrate (50% protein; Isochill 6000; Agropur Dairy Cooperative, St-Hubert, QC, Canada). ^2^ Whey protein concentrate (80% protein). ^3^ WHye protein concentrate (34% protein). ^4^ Contained 7.8% lysolecithin.

**Table 2:** Fatty acid profiles of experimental milk replacers (MR) and diets.

	MR			Diet	
	CO	PO	SO	PD	SD
FA % of DM	15.30	15.43	16.39	6.32	7.65
FA profile % of total FA					
8:0	1.56	2.04	1.41	0.61	0.08
10:0	1.46	2.46	1.34	1.52	0.11
12:0	9.93	12.14	8.79	1.94	0.36
14:0	5.75	9.55	5.31	6.59	0.44
14:1	0.10	0.38	0.11	0.54	0.06
15:0	0.16	0.59	0.18	0.83	0.08
16:0	21.79	25.22	20.99	26.53	13.59
16:1	1.74	1.49	1.60	1.15	0.23
17:0	0.27	0.37	0.27	0.43	0.12
18:0	10.60	9.98	9.97	8.13	3.41
18:1.*cis*-9	31.49	24.23	30.04	23.89	25.50
18:1.*cis*-11	2.21	1.79	2.10	1.31	1.20
18:2n6	10.24	6.90	14.40	22.51	48.62
18:3n6	0.09	0.10	0.08	0.13	0.05
18:3n3	0.43	0.39	1.05	0.99	4.60
20:0	0.19	0.18	0.21	0.27	0.38
20:4n6	0.17	0.22	0.16	0.25	0.03
22:00	0.05	0.07	0.09	0.18	0.30
22:5n3	0.07	0.07	0.08	0.08	0.03
22:6n3	0.04	0.06	0.04	0.09	0.01
Unidentified	1.65	1.78	1.79	2.02	0.79

Treatments were (1) milk replacer (MR), commercial milk substitute rich in animal fat and coconut oil (CO), milk substitute rich in polar lipids (PO), or milk substitute rich in soy lipids (SO); and (2) diet, solid feed containing soy lipids (SD) or lipids from cow milk fat globular membranes (PD). FA, fatty acid; DM, dry matter.

**Table 3:** Effects of milk replacers (MR) and diets on the concentration of plasma lipid classes in piglets on day 7 of the nursery phase.

Lipid Category	Lipid Class	Milk Replacer			Diet			*p*-Value		
		CO	PO	SO	PD	SD	SEM	MR	Diet	MR × Diet
Sphingolipids	Cer _µM_	2.43 ^a^	2.20 ^a^	1.87 ^b^	2.60 ^A^	1.78 ^B^	0.12	0.01	<0.0001	0.64
	HexCer _µM_	2.95 ^a^	2.56 ^ab^	2.38 ^b^	2.84 ^A^	2.42 ^B^	0.14	0.04	0.02	0.42
	Hex2Cer _µM_	0.94 ^a^	0.85 ^ab^	0.76 ^b^	0.89 ^A^	0.80 ^B^	0.03	0.002	0.02	0.94
	Hex3Cer _µM_	0.32 ^a^	0.31 ^ab^	0.28 ^b^	0.32 ^A^	0.29 ^B^	0.01	0.02	0.01	0.23
	SM(OH) _µM_	9.83 ^b^	10.92 ^a^	8.84 ^b^	10.55 ^A^	9.16 ^B^	0.47	0.01	0.02	0.56
	SM _µM_	133	129	114	135 ^A^	116 ^B^	6.33	0.09	0.01	0.64
Glycerolipids	DG _µM_	15.0	14.6	13.2	14.4	14.2	0.75	0.22	0.83	0.96
	TG _µM_	403	381	295	374	340	41	0.13	0.47	0.22
Phospholipids ^1^	PC aa _µM_	635 ^a^	644 ^a^	572 ^b^	650 ^A^	584 ^B^	20.8	0.03	0.01	0.83
	PC ae _µM_	67.0	71.3	64.4	69.1	66.0	2.87	0.27	0.37	0.60
	Lyso _µM_	84.1	84.3	78.7	82.6	82.0	3.32	0.39	0.88	0.66
Sterol lipids	CE _mM_	4.13 ^a^	3.86 ^a^	3.23 ^b^	3.99 ^A^	3.47 ^B^	0.19	0.004	0.02	0.45

Treatments were (1) milk replacer, commercial milk substitute rich in animal fat and coconut oil (CO), milk substitute rich in polar lipids (PO), or milk substitute rich in soy lipids (SO); and (2) diet, solid feed containing soy lipids (SD) or lipids from cow milk fat globular membranes (PD). Sphingolipids: ceramide (Cer); sphingomyelin (SM); sphingomyelin with a hydroxyl group (SM (OH)); ceramide with a hexose sugar residue attached to the sphingoid base and a fatty acid chain (HexCer); ceramide with two hexose sugar residues attached to the sphingoid base and a fatty acid chain (Hex2Cer); and ceramide with three hexose sugar residues attached to the sphingoid base and a fatty acid chain (Hex3Cer). Glycerol lipids: diacylglyceride (DG); triglyceride (TG). ^1^ Phospholipids containing choline: phosphatidylcholine with an acyl chain (PC aa); phosphatidylcholine with an acyl-alkyl chain (PC ae); and lysophosphatidylcholine (Lyso). Sterol lipids: Cholesteryl ester (CE). Values followed by different uppercase letters indicate significant differences between dietary treatments, while different lowercase letters indicate significant differences between MR treatments (*p* < 0.05).

## Data Availability

Available at Larsen, Rayllana (2024). Original data for Modulation of the Plasma Lipidomic Profile in Piglets Fed Polar Lipid-Rich Diets. figshare. Dataset. https://doi.org/10.6084/m9.figshare.27891141.v1 [37].

## Supplementary Materials

The following supporting information can be downloaded at: https://www.mdpi.com/article/10.3390/metabo14120673/s1, Table S1: Lipid composition of milk replacer and diet treatments as percentage of total lipids; Table S2: Concentrations (µM/g) of diverse lipid types identified in experimental milk replacers and diets; Table S3: Lipids identified in the plasma of piglets (µM) and their grouping into classes for lipidome analysis.
