# Supplementary material for: Modulation of the Plasma Lipidomic Profile in Piglets Fed Polar Lipid-Rich Diets
Source: Metabolites. 2024 Dec 3;14(12):673. doi: 10.3390/metabo14120673 (PMC11680061; doi:10.3390/metabo14120673)
Supplement: Supplementary file 1 [file metabolites-14-00673-s001.zip › metabolites-3244653-supplementary.pdf]

SUPPLEMENTAL MATERIAL

Supplemental Table S1. Lipid composition of milk replacer and diet treatments as percentage of total lipids.

Lipid category	Lipid class, % of total	Milk Replacer			Diet	
		CO	PO	SO	PD	SD
Sphingolipids	Cer	0.59	1.44	0.09	0.98	0.03
	HexCer	1.44	3.04	0.23	1.97	0.1
	Hex2Cer	0.65	1.21	0.14	1.0	0.02
	Hex3Cer	0.01	0.01	0.05	0.01	0.01
	SM	7.48	14.4	0.1	8.72	0.21
	SM (OH)	3.43	7.0	0.07	4.23	0.18
	**Total**	**13.6**	**27.1**	**0.68**	**16.91**	**0.55**
Glycerolipids	DG	6.57	8.71	21.4	9.45	24.6
	TG	49.6	21.7	75.4	43.7	44.1
	**Total**	**56.17**	**30.41**	**96.8**	**53.15**	**68.7**
Phospholipids	PC aa	23	30.8	0.61	23	17.5
	PC ae	3.75	5.61	0.24	4.03	0.58
	Lyso	3.29	3.13	0.43	2.52	12.5
	**Total**	**30.04**	**39.54**	**1.28**	**29.55**	**30.58**
Sterol lipids	CE	0.26	2.96	1.31	0.4	0.08

Treatments were: 1) Milk Replacer: commercial milk substitute rich in animal fat and coconut oil (CO); milk substitute rich in polar lipids (PO) or milk substitute rich in soy lipids (SO); 2) Diet: solid feed containing soy lipids (SD) or lipids from cow milk fat globular membranes (PD). Sphingolipids: ceramide (Cer); sphingomyelin (SM); sphingomyelin with a hydroxyl group (SM (OH)); ceramide with a hexose sugar residue attached to the sphingoid base and a fatty acid chain (HexCer); ceramide with two hexose sugar residues attached to the sphingoid base and a fatty acid chain (Hex2Cer); ceramide with three hexose sugar residues attached to the sphingoid base and a fatty acid chain (Hex3Cer). Glycerol lipids: diacylglyceride (DG); triglyceride (TG). [1]Phospholipids containing choline: phosphatidylcholine with an acyl chain (PC aa); phosphatidylcholine with an acyl-alkyl chain (PC ae); lysophosphatidylcholine (Lyso). Sterol lipids: Cholesteryl ester (CE).

Supplemental Table S2. Concentrations (μM/g) of diverse lipid types identified in experimental Milk Replacers and Diets.

Category	Class	Lipid	Milk Replacer			Diet	
			CO	PO	SO	PD	SD
Sphingolipids	Cer	Cer(d16:1/18:0)	0.011	0.145	0.011	0.076	0.011
		Cer(d16:1/20:0)	0.005	0.091	0.005	0.037	0.005
		Cer(d16:1/22:0)	0.516	1.741	0.002	0.898	0.029
		Cer(d16:1/24:0)	0.433	1.528	0.004	0.781	0.004
		Cer(d18:0/20:0)	0.005	0.024	0.005	0.005	0.005
		Cer(d18:0/22:0)	0.050	0.098	0.003	0.096	0.002
		Cer(d18:0/24:0)	0.002	0.084	0.002	0.027	0.047
		Cer(d18:0/24:1)	0.002	0.017	0.003	0.018	0.002
		Cer(d18:1/16:0)	1.394	5.019	0.010	2.266	0.068
		Cer(d18:1/18:0)	0.148	0.609	0.002	0.303	0.002
		Cer(d18:1/22:0)	1.024	3.693	0.004	2.025	0.086
		Cer(d18:1/23:0)	1.091	3.951	0.009	1.810	0.081
		Cer(d18:1/24:0)	0.687	2.704	0.003	1.149	0.003
		Cer(d18:1/24:1)	0.139	0.495	0.007	0.261	0.007
		Cer(d18:1/25:0)	0.024	0.174	0.024	0.024	0.024
		Cer(d18:2/22:0)	0.035	0.090	0.002	0.065	0.034
		Cer(d18:2/24:0)	0.041	0.049	0.003	0.045	0.002
		Cer(d18:2/24:1)	0.003	0.003	0.003	0.003	0.003
	HexCer	HexCer(d16:1/22:0)	1.098	2.881	0.068	1.466	0.087
		HexCer(d18:1/16:0)	3.251	10.519	0.006	4.524	0.060
		HexCer(d18:1/18:0)	0.402	1.323	0.006	0.592	0.050
		HexCer(d18:1/18:1)	0.112	0.279	0.004	0.121	0.031
		HexCer(d18:1/20:0)	0.265	0.780	0.017	0.319	0.032
		HexCer(d18:1/22:0)	3.066	10.508	0.022	4.588	0.024
		HexCer(d18:1/23:0)	2.683	9.343	0.015	4.326	0.044
		HexCer(d18:1/24:0)	1.836	5.833	0.047	2.775	0.158
		HexCer(d18:1/24:1)	0.440	0.913	0.021	0.662	0.333
		HexCer(d18:1/26:0)	0.059	0.088	0.007	0.053	0.051
		HexCer(d18:1/26:1)	0.028	0.047	0.021	0.038	0.045
		HexCer(d18:2/16:0)	0.084	0.252	0.002	0.099	0.057
		HexCer(d18:2/18:0)	0.046	0.043	0.004	0.032	0.038
		HexCer(d18:2/20:0)	0.015	0.020	0.002	0.017	0.031
		HexCer(d18:2/22:0)	0.092	0.182	0.003	0.079	0.019
		HexCer(d18:2/23:0)	0.061	0.168	0.002	0.083	0.033
		HexCer(d18:2/24:0)	0.060	0.159	0.004	0.070	0.102
	Hex2Cer	Hex2Cer(d18:1/14:0)	0.127	0.355	0.022	0.198	0.044
		Hex2Cer(d18:1/16:0)	2.163	6.992	0.037	3.529	0.071
		Hex2Cer(d18:1/18:0)	0.301	0.946	0.023	0.536	0.034
		Hex2Cer(d18:1/20:0)	0.222	0.532	0.016	0.316	0.042
		Hex2Cer(d18:1/22:0)	1.974	4.950	0.018	3.283	0.030
		Hex2Cer(d18:1/24:0)	1.139	2.840	0.015	1.883	0.015

		Hex2Cer(d18:1/24:1)	0.219	0.567	0.021	0.355	0.039
	Hex3Cer	Hex3Cer(d18:1/16:0)	0.003	0.029	0.003	0.003	0.016
		Hex3Cer(d18:1/18:0)	0.024	0.032	0.019	0.020	0.024
		Hex3Cer(d18:1/24:1)	0.061	0.055	0.031	0.059	0.042
		Hex3Cer(d18:1_22:0)	0.032	0.043	0.003	0.048	0.030
	SM(OH)	SM(OH) C14:1	7.316	20.043	0.022	7.835	0.073
		SM(OH) C16:1	3.317	8.817	0.006	3.738	0.076
		SM(OH) C22:1	17.477	60.099	0.035	25.659	0.754
		SM(OH) C22:2	2.749	6.466	0.004	3.207	1.248
		SM(OH) C24:1	1.501	4.222	0.004	2.215	0.124
	SM	SM C16:0	42.817	124.541	0.027	51.774	0.444
		SM C16:1	2.541	6.491	0.005	2.343	0.060
		SM C18:0	8.621	23.862	0.025	10.144	0.473
		SM C18:1	1.911	5.120	0.007	1.877	0.081
		SM C20:2	0.217	0.602	0.001	0.181	0.058
		SM C24:0	10.441	33.988	0.010	16.268	0.671
		SM C24:1	3.618	9.114	0.028	4.712	0.686
		SM C26:0	0.285	0.683	0.004	0.383	0.136
		SM C26:1	0.186	0.470	0.006	0.220	0.055
Glycerol lipids	DG	DG(14:0_18:1)	2.805	6.650	0.018	4.214	0.547
		DG(14:0_18:2)	1.141	1.454	0.208	1.202	1.088
		DG(14:0_20:0)	0.162	1.853	2.236	1.867	1.862
		DG(16:0_16:0)	12.267	58.498	2.025	27.259	4.925
		DG(16:0_16:1)	1.613	3.240	0.979	1.916	1.062
		DG(16:0_18:1)	11.183	18.489	1.190	18.040	16.260
		DG(16:0_18:2)	3.911	3.885	2.001	5.886	36.095
		DG(16:0_20:0)	0.251	0.502	0.190	0.344	0.437
		DG(16:1_18:1)	2.775	4.529	3.435	5.751	3.417
		DG(16:1_18:2)	0.822	0.776	0.297	0.838	1.150
		DG(16:1_20:0)	0.246	0.302	0.392	0.386	0.256
		DG(17:0_17:1)	0.144	0.254	0.281	0.241	0.285
		DG(17:0_18:1)	0.596	1.155	0.289	0.734	0.398
		DG(18:0_20:0)	0.034	0.189	0.034	0.034	0.175
		DG(18:1_18:1)	15.897	13.474	1.518	17.048	45.978
		DG(18:1_18:2)	4.403	3.296	1.468	4.551	58.500
		DG(18:1_18:3)	0.411	0.413	0.371	0.371	8.700
		DG(18:1_20:3)	0.072	0.172	0.049	0.083	0.086
		DG(18:1_20:4)	0.157	0.135	0.135	0.152	0.096
		DG(18:1_22:6)	0.965	3.460	0.861	2.013	0.903
		DG(18:2_18:2)	2.232	1.365	5.220	2.314	124.467
	TG	TG(14:0_32:2)	0.024	0.680	0.174	0.341	0.024
		TG(14:0_34:0)	0.263	0.582	0.261	0.177	0.012
		TG(14:0_34:1)	2.111	3.432	0.717	2.186	0.234
		TG(14:0_34:2)	1.031	1.331	0.381	1.207	0.050
		TG(14:0_34:3)	0.202	0.167	0.017	0.141	0.017
		TG(14:0_35:1)	0.119	0.264	0.176	0.022	0.165

TG(14:0_35:2)	0.149	0.028	0.028	0.160	0.028
TG(14:0_36:1)	1.221	1.129	0.077	1.173	0.005
TG(14:0_36:2)	3.460	2.695	0.024	3.132	0.024
TG(14:0_36:3)	1.864	1.433	0.060	1.811	0.210
TG(14:0_36:4)	0.455	0.417	0.032	0.544	0.280
TG(14:0_38:4)	0.052	0.046	0.006	0.042	0.006
TG(14:0_38:5)	0.006	0.006	0.006	0.006	0.006
TG(16:0_28:1)	0.953	4.303	0.037	1.285	0.020
TG(16:0_28:2)	0.344	1.191	0.029	0.544	0.165
TG(16:0_30:2)	0.427	1.262	0.217	0.799	0.015
TG(16:0_32:0)	1.446	0.453	2.467	1.151	0.951
TG(16:0_32:1)	2.569	4.376	2.176	3.027	0.762
TG(16:0_32:2)	0.833	0.866	0.095	0.322	0.009
TG(16:0_32:3)	0.095	0.203	0.012	0.099	0.076
TG(16:0_33:1)	0.471	0.357	1.529	0.770	0.423
TG(16:0_33:2)	0.023	0.190	0.217	0.023	0.023
TG(16:0_34:0)	0.994	0.470	0.992	0.556	0.228
TG(16:0_34:1)	11.767	9.396	1.831	11.326	4.185
TG(16:0_34:2)	7.120	4.713	1.132	7.265	8.121
TG(16:0_34:3)	1.189	0.637	0.131	1.018	0.788
TG(16:0_34:4)	0.029	0.028	0.030	0.055	0.018
TG(16:0_35:1)	0.581	0.533	0.451	0.610	0.092
TG(16:0_35:2)	0.297	0.254	0.237	0.388	0.010
TG(16:0_35:3)	0.207	0.148	0.070	0.214	0.093
TG(16:0_36:2)	27.929	15.917	0.308	24.287	6.713
TG(16:0_36:3)	16.500	8.761	1.143	14.801	18.002
TG(16:0_36:4)	4.153	2.453	1.958	4.401	25.817
TG(16:0_36:5)	0.283	0.212	0.501	0.382	5.244
TG(16:0_36:6)	0.050	0.007	0.045	0.007	0.505
TG(16:0_37:3)	0.142	0.108	0.052	0.190	0.877
TG(16:0_38:1)	0.203	0.095	0.117	0.172	0.102
TG(16:0_38:2)	0.746	0.397	0.033	0.636	0.367
TG(16:0_38:3)	0.843	0.416	0.027	0.853	0.444
TG(16:0_38:4)	0.473	0.389	0.012	0.513	0.121
TG(16:0_38:5)	0.205	0.119	0.008	0.204	0.008
TG(16:0_38:6)	0.087	0.015	0.015	0.080	0.015
TG(16:0_38:7)	0.126	0.137	0.160	0.186	0.004
TG(16:0_40:6)	0.064	0.009	0.009	0.071	0.009
TG(16:0_40:7)	0.089	0.088	0.107	0.099	0.076
TG(16:0_40:8)	0.061	0.061	0.017	0.073	0.012
TG(16:1_28:0)	0.299	0.137	0.541	0.392	0.197
TG(16:1_30:1)	0.657	0.676	1.141	0.686	0.494
TG(16:1_32:0)	0.648	0.687	2.242	1.034	0.950
TG(16:1_32:1)	0.686	1.009	2.205	0.998	0.907
TG(16:1_32:2)	0.115	0.009	0.895	0.009	0.009
TG(16:1_33:1)	1.088	0.873	1.292	1.178	0.542

TG(16:1_34:0)	0.815	0.692	0.359	0.850	0.131
TG(16:1_34:1)	4.461	2.763	1.202	4.340	0.500
TG(16:1_34:2)	2.170	1.138	1.039	1.774	0.226
TG(16:1_34:3)	0.417	0.129	0.158	0.430	0.015
TG(16:1_36:1)	1.377	0.741	0.276	1.310	0.025
TG(16:1_36:2)	4.103	2.148	0.430	3.767	0.196
TG(16:1_36:3)	2.491	1.419	0.030	2.095	0.426
TG(16:1_36:4)	0.700	0.396	0.033	0.688	0.491
TG(16:1_36:5)	0.011	0.011	0.026	0.057	0.101
TG(16:1_38:3)	0.177	0.071	0.060	0.110	0.006
TG(16:1_38:4)	0.126	0.064	0.019	0.067	0.074
TG(16:1_38:5)	0.035	0.033	0.011	0.045	0.095
TG(17:0_32:1)	0.439	0.276	0.661	0.316	0.092
TG(17:0_34:1)	0.431	0.459	0.453	0.490	0.153
TG(17:0_34:2)	0.171	0.180	0.205	0.226	0.115
TG(17:0_34:3)	0.014	0.014	0.018	0.014	0.014
TG(17:0_36:3)	0.415	0.380	0.077	0.438	0.305
TG(17:0_36:4)	0.108	0.078	0.034	0.126	0.405
TG(17:1_32:1)	0.296	0.372	0.664	0.375	0.141
TG(17:1_34:1)	0.407	0.326	0.410	0.433	0.188
TG(17:1_34:2)	0.303	0.169	0.136	0.312	0.130
TG(17:1_34:3)	0.019	0.019	0.019	0.019	0.019
TG(17:1_36:3)	0.255	0.161	0.043	0.300	0.150
TG(17:1_36:4)	0.093	0.065	0.022	0.084	0.157
TG(18:0_30:0)	0.083	0.515	0.351	0.129	0.085
TG(18:0_30:1)	0.562	0.840	0.234	0.508	0.012
TG(18:0_32:0)	0.369	0.325	0.529	0.398	0.116
TG(18:0_32:1)	2.505	1.659	0.310	2.055	0.006
TG(18:0_32:2)	0.855	0.411	0.096	0.721	0.003
TG(18:0_34:2)	7.691	4.504	0.308	7.126	1.585
TG(18:0_34:3)	0.923	0.604	0.027	0.942	0.223
TG(18:0_36:1)	1.134	0.840	0.143	1.084	0.329
TG(18:0_36:2)	4.038	2.419	0.340	3.581	1.803
TG(18:0_36:3)	3.145	1.864	0.381	2.985	4.062
TG(18:0_36:4)	1.361	0.601	0.398	1.277	5.106
TG(18:0_36:5)	0.117	0.074	0.116	0.126	1.032
TG(18:0_38:6)	0.010	0.010	0.010	0.010	0.010
TG(18:0_38:7)	0.048	0.008	0.008	0.057	0.008
TG(18:1_26:0)	5.189	9.350	0.048	5.740	0.333
TG(18:1_28:1)	2.323	3.180	0.208	2.333	0.252
TG(18:1_30:0)	3.056	4.885	0.948	2.998	0.232
TG(18:1_30:1)	2.785	3.401	0.609	2.861	0.012
TG(18:1_30:2)	0.817	1.051	0.074	0.925	0.176
TG(18:1_31:0)	0.344	0.245	1.153	0.464	0.442
TG(18:1_32:0)	9.917	7.617	1.143	8.857	1.495
TG(18:1_32:1)	14.241	9.489	0.993	13.073	0.191

TG(18:1_32:2)	3.552	2.382	0.057	3.248	0.007
TG(18:1_32:3)	0.318	0.295	0.042	0.338	0.150
TG(18:1_33:0)	0.532	0.387	0.501	0.637	0.235
TG(18:1_33:1)	0.889	0.498	0.723	0.631	0.272
TG(18:1_33:2)	0.237	0.221	0.202	0.222	0.017
TG(18:1_34:1)	77.931	44.944	0.274	72.173	9.259
TG(18:1_34:2)	35.171	18.784	0.832	31.656	15.689
TG(18:1_34:3)	4.555	2.369	0.268	4.337	2.484
TG(18:1_34:4)	0.281	0.209	0.020	0.261	0.251
TG(18:1_35:2)	1.134	0.695	0.121	1.260	0.271
TG(18:1_35:3)	0.339	0.223	0.064	0.365	0.197
TG(18:1_36:0)	1.169	0.693	0.088	1.168	0.243
TG(18:1_36:1)	8.868	5.374	0.523	7.876	3.595
TG(18:1_36:2)	25.866	13.233	1.896	22.320	22.822
TG(18:1_36:3)	16.018	9.209	2.180	15.022	35.611
TG(18:1_36:4)	5.333	2.876	2.434	5.285	36.885
TG(18:1_36:5)	0.564	0.345	0.735	0.781	8.343
TG(18:1_36:6)	0.118	0.095	0.096	0.209	0.884
TG(18:1_38:5)	0.185	0.116	0.025	0.156	0.048
TG(18:1_38:6)	0.058	0.051	0.003	0.097	0.022
TG(18:1_38:7)	0.187	0.114	0.036	0.186	0.128
TG(18:2_28:0)	1.075	1.283	0.109	1.006	0.071
TG(18:2_30:0)	0.943	1.456	0.324	0.960	0.003
TG(18:2_30:1)	1.087	1.004	0.259	0.832	0.021
TG(18:2_31:0)	0.230	0.094	0.283	0.143	0.077
TG(18:2_32:0)	3.685	2.565	0.416	3.741	3.392
TG(18:2_32:1)	4.503	2.842	0.346	3.858	0.049
TG(18:2_32:2)	1.255	0.955	0.107	1.382	0.991
TG(18:2_33:0)	0.316	0.210	0.211	0.273	0.012
TG(18:2_33:1)	0.122	0.273	0.319	0.424	0.075
TG(18:2_33:2)	0.016	0.105	0.034	0.109	0.169
TG(18:2_34:0)	8.482	4.489	0.260	7.924	2.271
TG(18:2_34:1)	27.986	15.168	0.996	25.242	17.800
TG(18:2_34:2)	13.263	7.040	2.862	12.296	39.633
TG(18:2_34:3)	1.779	0.999	0.434	1.559	4.625
TG(18:2_34:4)	0.124	0.015	0.034	0.098	0.284
TG(18:2_35:1)	0.693	0.454	0.121	0.752	0.706
TG(18:2_35:2)	0.566	0.279	0.071	0.587	0.877
TG(18:2_35:3)	0.183	0.031	0.053	0.193	0.340
TG(18:2_36:0)	0.594	0.373	0.051	0.540	0.467
TG(18:2_36:1)	3.878	2.334	0.432	3.487	4.489
TG(18:2_36:2)	10.044	5.477	1.728	9.364	25.031
TG(18:2_36:3)	7.975	4.473	4.320	7.590	62.158
TG(18:2_36:4)	2.794	1.667	7.463	3.407	89.672
TG(18:2_36:5)	0.345	0.148	1.976	0.555	18.831
TG(18:2_38:4)	0.201	0.144	0.017	0.262	0.293

TG(18:2_38:5)	0.095	0.016	0.016	0.115	0.091
TG(18:2_38:6)	0.035	0.004	0.006	0.055	0.034
TG(18:3_30:0)	0.075	0.123	0.013	0.009	0.009
TG(18:3_32:0)	0.183	0.108	0.031	0.238	0.363
TG(18:3_32:1)	0.329	0.181	0.015	0.288	0.113
TG(18:3_34:0)	0.420	0.293	0.027	0.424	0.355
TG(18:3_34:1)	1.660	1.003	0.217	1.361	2.886
TG(18:3_34:2)	0.697	0.382	0.475	0.684	4.780
TG(18:3_34:3)	0.139	0.111	0.085	0.158	0.935
TG(18:3_36:1)	0.302	0.178	0.057	0.355	0.777
TG(18:3_36:2)	0.896	0.591	0.326	0.817	4.314
TG(18:3_36:3)	0.763	0.554	0.809	0.879	9.148
TG(18:3_36:4)	0.215	0.203	1.257	0.407	11.281
TG(18:3_38:5)	0.010	0.010	0.010	0.010	0.010
TG(18:3_38:6)	0.009	0.009	0.009	0.009	0.009
TG(20:0_32:3)	0.012	0.012	0.012	0.012	0.088
TG(20:0_32:4)	0.024	0.024	0.035	0.024	0.195
TG(20:0_34:1)	0.202	0.964	1.391	1.274	1.096
TG(20:1_24:3)	0.011	0.011	0.011	0.011	0.011
TG(20:1_26:1)	0.011	0.053	0.011	0.011	0.011
TG(20:1_30:1)	0.081	0.066	0.007	0.057	0.007
TG(20:1_31:0)	0.059	0.068	0.011	0.066	0.011
TG(20:1_32:1)	0.192	0.077	0.016	0.144	0.011
TG(20:1_32:2)	0.016	0.016	0.320	0.016	0.016
TG(20:1_32:3)	0.043	0.006	0.006	0.006	0.006
TG(20:1_34:0)	0.105	0.142	0.008	0.209	0.006
TG(20:1_34:1)	0.996	0.677	0.058	0.970	0.282
TG(20:1_34:2)	0.615	0.322	0.052	0.600	0.428
TG(20:1_34:3)	0.111	0.013	0.013	0.107	0.075
TG(20:2_32:0)	0.136	0.134	0.022	0.122	0.022
TG(20:2_32:1)	0.140	0.111	0.023	0.144	0.017
TG(20:2_34:1)	0.970	0.494	0.009	0.741	0.009
TG(20:2_34:2)	0.476	0.287	0.017	0.407	0.140
TG(20:2_34:3)	0.085	0.011	0.015	0.083	0.087
TG(20:3_32:0)	0.023	0.023	0.023	0.023	0.023
TG(20:3_32:1)	0.018	0.018	0.018	0.093	0.018
TG(20:3_32:2)	0.003	0.003	0.244	0.003	0.003
TG(20:3_34:0)	0.123	0.101	0.007	0.090	0.007
TG(20:3_34:1)	0.421	0.197	0.014	0.318	0.014
TG(20:3_34:2)	0.228	0.137	0.013	0.218	0.086
TG(20:3_34:3)	0.052	0.036	0.033	0.005	0.121
TG(20:3_36:3)	0.112	0.087	0.009	0.143	0.074
TG(20:3_36:4)	0.061	0.035	0.003	0.082	0.039
TG(20:4_30:0)	0.030	0.054	0.016	0.014	0.002
TG(20:4_32:0)	0.071	0.066	0.007	0.094	0.007
TG(20:4_32:1)	0.099	0.013	0.013	0.013	0.013

	TG(20:4_32:2)	0.034	0.002	0.002	0.002	0.002
	TG(20:4_34:0)	0.128	0.088	0.007	0.112	0.007
	TG(20:4_34:1)	0.499	0.289	0.006	0.408	0.006
	TG(20:4_34:2)	0.211	0.182	0.014	0.275	0.014
	TG(20:4_34:3)	0.106	0.062	0.012	0.176	0.012
	TG(20:4_36:2)	0.262	0.185	0.004	0.219	0.004
	TG(20:4_36:3)	0.245	0.305	0.013	0.477	0.013
	TG(20:4_36:4)	0.172	0.270	0.003	0.253	0.021
	TG(20:5_34:0)	0.014	0.079	0.014	0.014	0.014
	TG(20:5_34:1)	0.248	0.157	0.016	0.306	0.016
	TG(20:5_34:2)	0.426	0.305	0.008	0.609	0.074
	TG(20:5_36:2)	0.180	0.144	0.043	0.162	0.056
	TG(20:5_36:3)	0.181	0.091	0.014	0.264	0.158
	TG(22:4_34:2)	0.079	0.020	0.002	0.059	0.068
	TG(22:5_32:0)	0.158	0.116	0.180	0.064	0.059
	TG(22:5_32:1)	0.805	0.436	0.021	0.600	0.209
	TG(22:5_34:1)	0.270	0.154	0.006	0.275	0.219
	TG(22:5_34:2)	0.143	0.069	0.012	0.167	0.418
	TG(22:6_32:0)	0.013	0.013	0.013	0.104	0.013
	TG(22:6_32:1)	0.029	0.005	0.048	0.047	0.005
	TG(22:6_34:1)	0.171	0.240	0.008	0.248	0.159
	TG(22:6_34:2)	0.130	0.163	0.010	0.132	0.289
[1]PhospholipidsPC aa	PC aa C24:0	0.691	1.392	0.015	0.559	0.015
	PC aa C26:0	0.128	0.128	0.128	0.128	0.128
	PC aa C28:1	2.636	5.725	0.006	2.586	0.059
	PC aa C30:0	17.048	43.818	0.022	21.638	0.133
	PC aa C32:0	16.370	46.097	0.013	23.752	0.639
	PC aa C32:1	13.146	27.762	0.012	13.453	0.456
	PC aa C32:2	1.800	3.577	0.015	1.576	0.278
	PC aa C32:3	0.815	1.758	0.004	0.660	0.078
	PC aa C34:1	47.151	80.143	0.087	51.005	17.371
	PC aa C34:2	27.601	51.863	0.058	26.725	44.967
	PC aa C34:3	3.030	7.273	0.011	2.810	3.754
	PC aa C34:4	0.413	0.925	0.008	0.313	0.150
	PC aa C36:0	2.379	5.507	0.032	3.144	0.306
	PC aa C36:1	15.193	33.829	0.025	19.394	2.913
	PC aa C36:2	31.317	53.265	0.024	30.206	22.955
	PC aa C36:3	19.726	37.346	0.026	16.667	47.944
	PC aa C36:4	6.263	13.642	0.029	5.523	61.652
	PC aa C36:5	0.791	2.071	0.012	0.771	10.473
	PC aa C36:6	0.276	0.645	0.007	0.317	0.893
	PC aa C38:0	0.475	1.086	0.007	0.606	0.120
	PC aa C38:1	2.530	6.445	0.009	3.741	0.327
	PC aa C38:3	1.033	2.043	0.005	1.049	0.804
	PC aa C38:4	1.894	3.825	0.014	1.644	0.312
	PC aa C38:5	2.039	4.532	0.015	1.627	0.173

	PC aa C38:6	0.731	1.738	0.011	0.563	0.088
	PC aa C40:1	0.117	0.224	0.004	0.148	0.066
	PC aa C40:2	0.093	0.180	0.003	0.125	0.124
	PC aa C40:3	0.094	0.258	0.007	0.107	0.040
	PC aa C40:4	0.180	0.397	0.004	0.176	0.039
	PC aa C40:5	0.291	0.628	0.005	0.278	0.073
	PC aa C40:6	0.383	0.860	0.009	0.323	0.139
	PC aa C42:0	0.039	0.070	0.007	0.052	0.007
	PC aa C42:1	0.010	0.072	0.010	0.010	0.010
	PC aa C42:2	0.033	0.059	0.005	0.034	0.056
	PC aa C42:4	0.024	0.045	0.004	0.029	0.020
	PC aa C42:5	0.034	0.082	0.004	0.039	0.036
	PC aa C42:6	0.037	0.074	0.007	0.042	0.053
PC ae	PC ae C30:0	1.401	3.494	0.019	1.545	0.019
	PC ae C30:1	1.419	3.249	0.007	1.528	0.049
	PC ae C30:2	0.098	0.186	0.006	0.082	0.006
	PC ae C32:1	1.216	2.659	0.007	1.270	0.042
	PC ae C32:2	0.468	0.966	0.008	0.419	0.008
	PC ae C34:0	2.476	6.197	0.015	3.333	0.015
	PC ae C34:1	4.168	9.127	0.007	4.484	0.157
	PC ae C34:2	1.829	3.733	0.007	1.671	0.275
	PC ae C34:3	0.801	1.648	0.005	0.705	0.177
	PC ae C36:0	2.160	5.067	0.010	2.850	0.157
	PC ae C36:1	6.674	15.313	0.009	8.251	0.771
	PC ae C36:2	2.324	4.491	0.004	2.354	0.618
	PC ae C36:3	0.728	1.526	0.003	0.605	0.243
	PC ae C36:4	0.337	0.752	0.005	0.297	0.124
	PC ae C36:5	0.167	0.336	0.006	0.141	0.037
	PC ae C38:0	0.743	1.826	0.005	0.933	0.071
	PC ae C38:1	4.192	10.889	0.007	5.708	0.318
	PC ae C38:2	1.486	2.798	0.008	1.496	0.849
	PC ae C38:3	0.572	1.077	0.006	0.559	1.644
	PC ae C38:4	0.239	0.486	0.007	0.262	0.702
	PC ae C38:5	0.158	0.349	0.003	0.162	0.127
	PC ae C38:6	0.277	0.623	0.008	0.325	0.084
	PC ae C40:1	0.425	1.024	0.003	0.569	0.132
	PC ae C40:2	0.221	0.404	0.007	0.263	0.108
	PC ae C40:3	0.158	0.270	0.005	0.169	0.076
	PC ae C40:4	0.119	0.205	0.005	0.109	0.064
	PC ae C40:5	0.108	0.228	0.002	0.096	0.041
	PC ae C40:6	0.110	0.243	0.007	0.123	0.054
	PC ae C42:0	0.008	0.076	0.008	0.008	0.008
	PC ae C42:1	0.066	0.139	0.006	0.070	0.031
	PC ae C42:2	0.055	0.115	0.008	0.063	0.046
	PC ae C42:3	0.057	0.096	0.009	0.055	0.009
	PC ae C42:4	0.051	0.082	0.003	0.048	0.046

		PC ae C42:5	0.050	0.102	0.005	0.053	0.060
		PC ae C44:3	0.007	0.042	0.007	0.007	0.007
		PC ae C44:4	0.004	0.042	0.004	0.004	0.042
		PC ae C44:5	0.004	0.045	0.004	0.023	0.043
		PC ae C44:6	0.008	0.054	0.008	0.008	0.008
	Lyso	LysoPC a C14:0	6.315	6.494	0.026	5.087	0.670
		LysoPC a C16:0	10.218	10.181	0.125	8.479	21.484
		LysoPC a C16:1	0.369	0.577	0.015	0.279	0.683
		LysoPC a C17:0	0.393	0.514	0.005	0.338	0.148
		LysoPC a C18:0	2.106	3.490	0.011	2.056	2.587
		LysoPC a C18:1	4.247	7.391	0.061	3.097	43.505
		LysoPC a C18:2	1.722	3.154	0.065	1.365	86.001
		LysoPC a C20:3	0.018	0.018	0.018	0.018	0.018
		LysoPC a C20:4	0.203	0.390	0.006	0.148	0.079
		LysoPC a C24:0	0.215	0.498	0.007	0.184	0.068
		LysoPC a C26:0	1.489	3.218	0.026	1.160	0.277
		LysoPC a C26:1	0.245	0.470	0.009	0.208	0.113
		LysoPC a C28:0	2.238	5.511	0.079	1.875	0.235
		LysoPC a C28:1	1.261	2.667	0.010	1.105	0.147
Sterol lipids	CE	CE(14:0)	1.027	11.935	0.416	2.457	0.047
		CE(14:1)	0.048	0.048	0.048	0.048	0.048
		CE(15:0)	0.036	0.036	0.036	0.036	0.036
		CE(15:1)	0.015	0.015	0.015	0.015	0.015
		CE(16:0)	0.087	20.994	0.087	0.087	0.087
		CE(16:1)	0.119	0.119	0.119	0.119	0.119
		CE(17:0)	0.025	0.025	0.025	0.025	0.025
		CE(17:1)	0.051	0.051	0.051	0.051	0.051
		CE(18:0)	0.046	5.347	0.046	0.486	0.046
		CE(18:1)	0.074	0.074	0.074	0.074	0.074
		CE(18:2)	0.102	0.102	0.102	0.102	0.102
		CE(18:3)	0.044	0.044	0.044	0.044	0.044
		CE(20:0)	0.052	0.052	0.052	0.052	0.052
		CE(20:1)	0.029	0.029	0.029	0.029	0.029
		CE(20:3)	0.022	0.022	0.022	0.022	0.022
		CE(20:4)	0.022	0.022	0.022	0.022	0.022
		CE(20:5)	0.103	0.103	0.103	0.103	0.103
		CE(22:0)	0.046	0.046	0.046	0.046	0.046
		CE(22:1)	0.027	0.027	0.027	0.027	0.027
		CE(22:2)	0.019	0.019	0.019	0.019	0.019
		CE(22:5)	0.437	2.999	0.023	0.140	0.023
		CE(22:6)	0.013	0.013	0.013	0.013	0.013

Treatments were: 1) Milk Replacer: commercial milk substitute rich in animal fat and coconut oil (CO); milk substitute rich in polar lipids (PO) or milk substitute rich in soy lipids (SO); 2) Diet: solid feed containing soy lipids (SD) or lipids from cow milk fat globular membranes (PD). Sphingolipids: ceramide (Cer); ceramide with a hexose sugar residue attached to the sphingoid base and fatty acid chains (HexCer); ceramide with two hexose sugar residues attached to the sphingoid base and fatty acid chains

(Hex2Cer); ceramide with three hexose sugar residues attached to the sphingoid base and fatty acid chains (Hex3Cer); sphingomyelin (SM); sphingomyelin with a hydroxyl group (SM (OH)). Glycerolipids category: diacylglyceride (DG); triglyceride (TG). [1]Phospholipids containing choline: phosphatidylcholine with an acyl chain (PC aa); phosphatidylcholine with an acyl-alkyl chain (PC ae); lysophosphatidylcholine (Lyso). Sterol lipids category: Cholesteryl ester (CE).

Supplemental Table S3. Lipids identified in the plasma of piglets (μM) and their grouping into classes for lipidome analysis

Category	Class	Lipid	Mean	SD	Min.	Max.
		Cer(d16:1/18:0)	0.01	0	0.01	0.01
		Cer(d16:1/20:0)	0.04	0.02	0.005	0.1
		Cer(d16:1/22:0)	0.03	0.02	0.003	0.08
		Cer(d16:1/24:0)	0.04	0.03	0.004	0.12
		Cer(d18:1/16:0)	0.41	0.13	0.22	0.82
		Cer(d18:1/18:0)	0.12	0.06	0.02	0.3
		Cer(d18:1/22:0)	0.24	0.06	0.16	0.39
		Cer(d18:1/23:0)	0.19	0.1	0.07	0.46
		Cer(d18:1/24:0)	0.53	0.16	0.29	0.86
	Cer	Cer(d18:1/24:1)	0.44	0.14	0.22	0.78
		Cer(d18:1/25:0)	0.03	0.02	0.02	0.13
		Cer(d18:2/22:0)	0.03	0.02	0.01	0.1
		Cer(d18:2/24:0)	0.08	0.03	0.04	0.17
		Cer(d18:2/24:1)	0.06	0.03	0.02	0.15
		Cer(d18:0/20:0)	0.01	0.01	0.005	0.06
		Cer(d18:0/22:0)	0.01	0.01	0.002	0.05
Sphingolipids		Cer(d18:0/24:0)	0.01	0.01	0.002	0.06
		Cer(d18:0/24:1)	0	0	0.002	0.02
		HexCer(d16:1/22:0)	0.09	0.02	0.06	0.13
		HexCer(d18:1/16:0)	0.38	0.09	0.25	0.75
		HexCer(d18:1/18:0)	0.08	0.02	0.03	0.14
		HexCer(d18:1/18:1)	0.03	0.01	0.004	0.06
		HexCer(d18:1/20:0)	0.1	0.03	0.05	0.18
		HexCer(d18:1/22:0)	0.22	0.06	0.15	0.43
		HexCer(d18:1/23:0)	0.15	0.05	0.09	0.33
	HexCer	HexCer(d18:1/24:0)	0.65	0.17	0.4	1.22
		HexCer(d18:1/24:1)	0.62	0.62	0.34	4.72
		HexCer(d18:1/26:0)	0.06	0.01	0.01	0.09
		HexCer(d18:1/26:1)	0.04	0.01	0.02	0.08
		HexCer(d18:2/16:0)	0.03	0.01	0.01	0.05
		HexCer(d18:2/18:0)	0.02	0.01	0.003	0.05
		HexCer(d18:2/20:0)	0.04	0.02	0.01	0.1
		HexCer(d18:2/22:0)	0.05	0.02	0.01	0.13

		HexCer(d18:2/23:0)	0.04	0.02	0.002	0.12
		HexCer(d18:2/24:0)	0.13	0.05	0.06	0.29
	Hex2Cer	Hex2Cer(d18:1/14:0)	0.05	0.01	0.02	0.08
		Hex2Cer(d18:1/16:0)	0.32	0.07	0.21	0.49
		Hex2Cer(d18:1/18:0)	0.17	0.04	0.09	0.3
		Hex2Cer(d18:1/20:0)	0.06	0.01	0.04	0.09
		Hex2Cer(d18:1/22:0)	0.07	0.02	0.03	0.12
		Hex2Cer(d18:1/24:0)	0.08	0.04	0.02	0.14
		Hex2Cer(d18:1/24:1)	0.12	0.03	0.07	0.19
	Hex3Cer	Hex3Cer(d18:1/16:0)	0.13	0.03	0.07	0.19
		Hex3Cer(d18:1/18:0)	0.04	0.01	0.02	0.07
		Hex3Cer(d18:1/24:1)	0.11	0.02	0.07	0.16
		Hex3Cer(d18:1_22:0)	0.03	0.01	0.003	0.07
	SM(OH)	SM(OH) C14:1	2.02	0.72	1.09	4.17
		SM(OH) C16:1	2.09	0.44	1.43	3.38
		SM(OH) C22:2	2.37	0.55	1.48	3.69
		SM(OH) C22:1	3.19	0.74	1.99	5.03
		SM(OH) C24:1	0.41	0.07	0.28	0.62
	SM	SM C16:1	5.48	1.28	3.5	9.07
		SM C16:0	65.1	13.5	46.3	105
		SM C18:1	4.7	1.36	2.28	7.97
		SM C18:0	16.36	4.93	8.46	27.3
		SM C20:2	0.18	0.04	0.13	0.28
		SM C24:1	25.8	6.21	15.9	43.3
		SM C24:0	9.99	2.11	6.73	16.4
		SM C26:1	0.51	0.12	0.31	0.8
		SM C26:0	0.19	0.03	0.14	0.31
Glycerol lipids	DG	DG(14:0_18:1)	0.09	0.12	0.02	0.71
		DG(14:0_18:2)	0.19	0.06	0.09	0.37
		DG(14:0_20:0)	0.75	0.39	0.13	1.61
		DG(16:0_16:0)	3.6	1.49	2.18	11.67
		DG(16:0_16:1)	0.79	0.35	0.42	2.94
		DG(16:0_18:1)	1.2	0.41	0.66	3.09
		DG(16:0_18:2)	0.77	0.22	0.4	1.48
		DG(16:0_20:0)	0.44	0.08	0.32	0.74
		DG(16:1_18:1)	1.61	0.65	0.45	3.06
		DG(16:1_18:2)	0.34	0.09	0.2	0.77
		DG(16:1_20:0)	0.26	0.06	0.16	0.58
		DG(17:0_17:1)	0.16	0.04	0.09	0.39
		DG(17:0_18:1)	0.27	0.11	0.15	0.91
		DG(18:0_20:0)	0.04	0.03	0.03	0.19
		DG(18:1_18:1)	1.41	0.43	0.77	3.2
		DG(18:1_18:2)	0.96	0.32	0.55	1.93
		DG(18:1_18:3)	0.15	0.04	0.07	0.26
		DG(18:1_20:3)	0.09	0.02	0.04	0.16
		DG(18:1_20:4)	0.17	0.03	0.09	0.22

	DG(18:1_22:6)	0.38	0.15	0.12	0.69
	DG(18:2_18:2)	0.92	0.51	0.34	2.15
TG	TG(14:0_32:2)	0.63	0.67	0.02	2.51
	TG(14:0_34:0)	0.26	0.35	0.01	1.34
	TG(14:0_34:1)	2.07	2.01	0.02	7.76
	TG(14:0_34:2)	1.96	1.59	0.05	6.87
	TG(14:0_34:3)	0.38	0.28	0.02	1.11
	TG(14:0_35:1)	0.1	0.18	0.02	0.98
	TG(14:0_35:2)	0.1	0.14	0.03	0.71
	TG(14:0_36:1)	0.46	0.46	0.01	2.02
	TG(14:0_36:2)	1.69	1.34	0.27	5.93
	TG(14:0_36:3)	2.02	1.24	0.45	6.27
	TG(14:0_36:4)	1.02	0.53	0.25	2.67
	TG(14:0_38:4)	0.08	0.06	0.01	0.33
	TG(14:0_38:5)	0.08	0.06	0.01	0.25
	TG(16:0_28:1)	0.62	0.71	0.02	2.56
	TG(16:0_28:2)	0.28	0.29	0.03	1.03
	TG(16:0_30:2)	1.23	1.22	0.02	7.14
	TG(16:0_32:0)	1.46	2.05	0.03	9.29
	TG(16:0_32:1)	3.6	3.53	0.04	13.3
	TG(16:0_32:2)	2.21	1.97	0.01	8.13
	TG(16:0_32:3)	0.3	0.23	0.01	1
	TG(16:0_33:1)	0.5	1.04	0.01	6.32
	TG(16:0_33:2)	0.45	0.55	0.02	2.88
	TG(16:0_34:0)	0.85	0.99	0.01	4.4
	TG(16:0_34:1)	9.8	7.04	1.61	35.6
	TG(16:0_34:2)	12.5	7.04	3.2	36.7
	TG(16:0_34:3)	3.11	1.96	0.94	10.9
	TG(16:0_34:4)	0.36	0.25	0.08	1.33
	TG(16:0_35:1)	0.3	0.41	0.01	2.16
	TG(16:0_35:2)	0.45	0.43	0.01	1.81
	TG(16:0_35:3)	0.26	0.17	0.01	0.85
	TG(16:0_36:2)	11.8	6.84	3.28	39.7
	TG(16:0_36:3)	17.7	8.69	4.91	49.4
	TG(16:0_36:4)	10.3	5.65	2.57	24.3
	TG(16:0_36:5)	1.68	1.05	0.39	4.31
	TG(16:0_36:6)	0.11	0.09	0.01	0.37
	TG(16:0_37:3)	0.15	0.11	0.01	0.46
	TG(16:0_38:1)	0.08	0.11	0.01	0.64
	TG(16:0_38:2)	0.16	0.2	0.03	1.06
	TG(16:0_38:3)	0.41	0.26	0.08	1.54
	TG(16:0_38:4)	0.67	0.48	0.21	2.66
	TG(16:0_38:5)	0.97	0.69	0.27	3.37
	TG(16:0_38:6)	0.6	0.35	0.2	1.95
	TG(16:0_38:7)	0.06	0.08	0.004	0.33
	TG(16:0_40:6)	0.42	0.3	0.13	1.67

TG(16:0_40:7)	0.2	0.18	0.01	1.03
TG(16:0_40:8)	0.1	0.08	0.01	0.32
TG(16:1_28:0)	0.31	0.56	0.01	3.47
TG(16:1_30:1)	0.45	0.98	0.01	6.01
TG(16:1_32:0)	0.78	1.46	0.02	8.89
TG(16:1_32:1)	1.22	1.91	0.04	11.3
TG(16:1_32:2)	0.53	0.89	0.01	4.13
TG(16:1_33:1)	0.41	1.07	0.004	6.8
TG(16:1_34:0)	0.39	0.45	0.003	2.37
TG(16:1_34:1)	2.77	2.42	0.01	9.94
TG(16:1_34:2)	2.87	2.08	0.43	9.92
TG(16:1_34:3)	0.69	0.52	0.01	2.34
TG(16:1_36:1)	0.48	0.4	0.03	1.78
TG(16:1_36:2)	2.21	1.26	0.56	6.01
TG(16:1_36:3)	2.63	1.23	0.81	7.05
TG(16:1_36:4)	1.42	0.62	0.53	3.8
TG(16:1_36:5)	0.27	0.13	0.09	0.75
TG(16:1_38:3)	0.11	0.08	0.01	0.46
TG(16:1_38:4)	0.22	0.13	0.07	0.67
TG(16:1_38:5)	0.29	0.18	0.08	0.76
TG(17:0_32:1)	0.33	0.56	0.01	3.53
TG(17:0_34:1)	0.29	0.39	0.005	2.07
TG(17:0_34:2)	0.29	0.3	0.01	1.44
TG(17:0_34:3)	0.1	0.08	0.01	0.35
TG(17:0_36:3)	0.5	0.32	0.12	1.55
TG(17:0_36:4)	0.27	0.2	0.05	0.99
TG(17:1_32:1)	0.24	0.46	0.01	2.84
TG(17:1_34:1)	0.25	0.32	0.004	1.84
TG(17:1_34:2)	0.21	0.25	0.02	1.22
TG(17:1_34:3)	0.05	0.06	0.02	0.26
TG(17:1_36:3)	0.25	0.14	0.05	0.74
TG(17:1_36:4)	0.15	0.08	0.01	0.42
TG(18:0_30:0)	0.31	0.44	0.01	2.01
TG(18:0_30:1)	0.6	0.84	0.01	5.25
TG(18:0_32:0)	0.27	0.37	0.01	1.6
TG(18:0_32:1)	0.78	0.71	0.01	2.77
TG(18:0_32:2)	0.43	0.51	0.003	2.31
TG(18:0_34:2)	2.73	1.49	0.82	8.69
TG(18:0_34:3)	0.62	0.29	0.21	1.78
TG(18:0_36:1)	0.46	0.38	0.01	1.82
TG(18:0_36:2)	2.21	1.33	0.66	7.53
TG(18:0_36:3)	3.77	1.95	1.1	11.2
TG(18:0_36:4)	2.61	1.64	0.68	7.83
TG(18:0_36:5)	0.42	0.31	0.09	1.29
TG(18:0_38:6)	0.2	0.11	0.09	0.73
TG(18:0_38:7)	0.03	0.03	0.01	0.12

TG(18:1_26:0)	1.38	1.68	0.003	9.47
TG(18:1_28:1)	0.7	0.79	0.01	4.08
TG(18:1_30:0)	2.92	3.03	0.01	13.9
TG(18:1_30:1)	3.11	4.07	0.01	25.9
TG(18:1_30:2)	1.59	1.91	0.09	12
TG(18:1_31:0)	0.44	0.79	0.01	4.58
TG(18:1_32:0)	5.32	4.18	0.51	20.4
TG(18:1_32:1)	5.88	4.18	0.75	19.1
TG(18:1_32:2)	2.76	1.85	0.22	9.02
TG(18:1_32:3)	0.39	0.22	0.08	1.01
TG(18:1_33:0)	0.32	0.47	0.003	2.36
TG(18:1_33:1)	0.65	0.83	0.01	4.03
TG(18:1_33:2)	0.4	0.39	0.02	1.74
TG(18:1_34:1)	19	11.5	4.7	61.2
TG(18:1_34:2)	19.2	9.3	5.3	54.7
TG(18:1_34:3)	3.8	1.63	1.19	9.37
TG(18:1_34:4)	0.48	0.25	0.13	1.43
TG(18:1_35:2)	0.61	0.46	0.01	2.24
TG(18:1_35:3)	0.27	0.16	0.05	0.82
TG(18:1_36:0)	0.38	0.32	0.01	1.72
TG(18:1_36:1)	3.58	2.24	0.82	11.9
TG(18:1_36:2)	12.8	7.15	2.44	41.1
TG(18:1_36:3)	17.3	10.2	3.7	55.1
TG(18:1_36:4)	11.1	8.59	1.99	35.8
TG(18:1_36:5)	2.21	1.87	0.36	7.02
TG(18:1_36:6)	0.18	0.14	0.01	0.55
TG(18:1_38:5)	1.18	0.56	0.59	3.56
TG(18:1_38:6)	0.71	0.27	0.35	1.72
TG(18:1_38:7)	0.15	0.08	0.02	0.44
TG(18:2_28:0)	1.8	1.8	0.04	10.2
TG(18:2_30:0)	2.25	1.88	0.15	8.41
TG(18:2_30:1)	1.96	2.1	0.23	13.3
TG(18:2_31:0)	0.34	0.42	0.003	1.88
TG(18:2_32:0)	5.33	3.12	1.16	17
TG(18:2_32:1)	4.52	2.68	1.11	14.3
TG(18:2_32:2)	2.11	1.07	0.42	5.48
TG(18:2_33:0)	0.28	0.3	0.01	1.27
TG(18:2_33:1)	0.54	0.44	0.01	1.83
TG(18:2_33:2)	0.35	0.23	0.02	0.97
TG(18:2_34:0)	3.07	1.65	0.92	9.62
TG(18:2_34:1)	17.3	8.6	4.8	49
TG(18:2_34:2)	17.2	8.8	4.3	40.9
TG(18:2_34:3)	3.02	1.41	0.85	6.9
TG(18:2_34:4)	0.35	0.17	0.11	0.94
TG(18:2_35:1)	0.53	0.36	0.04	1.82
TG(18:2_35:2)	0.61	0.41	0.1	2.07

TG(18:2_35:3)	0.21	0.17	0.03	0.71
TG(18:2_36:0)	0.49	0.27	0.13	1.48
TG(18:2_36:1)	3.76	1.97	1.02	10.9
TG(18:2_36:2)	12.3	7.51	2.59	38
TG(18:2_36:3)	16.9	14.2	2.64	58.3
TG(18:2_36:4)	12.1	12.5	1.23	43.8
TG(18:2_36:5)	2.34	2.49	0.21	8.7
TG(18:2_38:4)	0.73	0.33	0.28	2.1
TG(18:2_38:5)	0.98	0.4	0.43	2.34
TG(18:2_38:6)	0.62	0.28	0.2	1.34
TG(18:3_30:0)	0.2	0.16	0.01	0.79
TG(18:3_32:0)	0.49	0.28	0.11	1.57
TG(18:3_32:1)	0.57	0.37	0.16	2.11
TG(18:3_34:0)	0.33	0.16	0.07	0.9
TG(18:3_34:1)	2.32	1.22	0.66	7.35
TG(18:3_34:2)	2.18	1.25	0.6	5.99
TG(18:3_34:3)	0.42	0.21	0.12	1.12
TG(18:3_36:1)	0.48	0.28	0.13	1.48
TG(18:3_36:2)	1.78	1.17	0.41	5.54
TG(18:3_36:3)	2.4	2.07	0.41	8.01
TG(18:3_36:4)	1.6	1.64	0.13	5.8
TG(18:3_38:5)	0.2	0.1	0.07	0.52
TG(18:3_38:6)	0.12	0.06	0.01	0.25
TG(20:0_32:3)	0.03	0.04	0.01	0.15
TG(20:0_32:4)	0.03	0.04	0.02	0.18
TG(20:0_34:1)	0.14	0.24	0.02	1.16
TG(20:1_24:3)	0.01	0	0.01	0.01
TG(20:1_26:1)	0.01	0.01	0.01	0.05
TG(20:1_30:1)	0.05	0.07	0.01	0.38
TG(20:1_31:0)	0.04	0.05	0.01	0.25
TG(20:1_32:1)	0.09	0.12	0.01	0.56
TG(20:1_32:2)	0.32	0.56	0.02	2.65
TG(20:1_32:3)	0.02	0.02	0.01	0.12
TG(20:1_34:0)	0.04	0.04	0.01	0.2
TG(20:1_34:1)	0.18	0.15	0.01	0.81
TG(20:1_34:2)	0.2	0.12	0.02	0.72
TG(20:1_34:3)	0.03	0.03	0.01	0.15
TG(20:2_32:0)	0.08	0.1	0.02	0.46
TG(20:2_32:1)	0.11	0.11	0.02	0.5
TG(20:2_34:1)	0.32	0.2	0.08	1.14
TG(20:2_34:2)	0.27	0.15	0.02	0.88
TG(20:2_34:3)	0.07	0.05	0.01	0.26
TG(20:3_32:0)	0.18	0.17	0.02	0.79
TG(20:3_32:1)	0.24	0.21	0.02	0.84
TG(20:3_32:2)	0.01	0.05	0.004	0.35
TG(20:3_34:0)	0.11	0.09	0.01	0.46

	TG(20:3_34:1)	0.78	0.54	0.22	2.8
	TG(20:3_34:2)	0.81	0.48	0.27	2.47
	TG(20:3_34:3)	0.22	0.11	0.09	0.54
	TG(20:3_36:3)	0.73	0.3	0.35	1.87
	TG(20:3_36:4)	0.42	0.16	0.18	0.89
	TG(20:4_30:0)	0.15	0.11	0.02	0.54
	TG(20:4_32:0)	0.55	0.42	0.11	1.93
	TG(20:4_32:1)	0.72	0.55	0.01	2.2
	TG(20:4_32:2)	0.12	0.18	0.003	0.7
	TG(20:4_34:0)	0.44	0.29	0.13	1.48
	TG(20:4_34:1)	2.44	1.53	0.76	7.04
	TG(20:4_34:2)	2.6	1.43	0.92	6.96
	TG(20:4_34:3)	0.67	0.36	0.21	1.55
	TG(20:4_36:2)	2.11	0.94	1.04	6.03
	TG(20:4_36:3)	2.39	0.94	1.06	5.46
	TG(20:4_36:4)	1.48	0.63	0.47	3.09
	TG(20:5_34:0)	0.03	0.04	0.01	0.19
	TG(20:5_34:1)	0.19	0.13	0.02	0.6
	TG(20:5_34:2)	0.2	0.11	0.06	0.52
	TG(20:5_36:2)	0.17	0.08	0.03	0.45
	TG(20:5_36:3)	0.24	0.11	0.06	0.49
	TG(22:4_34:2)	0.32	0.22	0.12	1.29
	TG(22:5_32:0)	0.13	0.17	0.004	0.74
	TG(22:5_32:1)	0.02	0	0.02	0.02
	TG(22:5_34:1)	0.5	0.38	0.09	1.94
	TG(22:5_34:2)	0.46	0.29	0.15	1.56
	TG(22:6_32:0)	0.05	0.07	0.01	0.34
	TG(22:6_32:1)	0.04	0.06	0.005	0.27
	TG(22:6_34:1)	0.23	0.13	0.08	0.71
	TG(22:6_34:2)	0.25	0.11	0.1	0.57
Phospholipids PC aa	PC aa C32:2	1.14	0.22	0.73	1.63
	PC aa C36:6	0.18	0.03	0.12	0.29
	PC aa C36:0	4.05	1.01	2.47	7.17
	PC aa C38:6	12.3	2.79	5.99	19
	PC aa C38:0	0.57	0.1	0.41	0.91
	PC aa C40:6	12.1	3.1	5.4	21.5
	PC aa C40:2	0.24	0.05	0.17	0.36
	PC aa C40:1	0.11	0.02	0.08	0.17
	PC aa C24:0	0.02	0.01	0.02	0.09
	PC aa C26:0	0.13	0	0.13	0.13
	PC aa C28:1	0.77	0.27	0.36	1.56
	PC aa C30:0	2.29	0.51	1.61	3.97
	PC aa C32:3	0.14	0.04	0.08	0.27
	PC aa C32:1	9.18	2.56	4.93	16.08
	PC aa C32:0	10.2	2.2	7.1	18.8
	PC aa C34:4	0.3	0.06	0.19	0.48

	PC aa C34:3	5.65	1.48	3.58	11.38
	PC aa C34:2	104	14.9	72.7	172
	PC aa C34:1	83.6	12.7	62.5	116
	PC aa C36:5	1.95	0.41	1.25	3.48
	PC aa C36:4	50.4	9.1	32.8	75.5
	PC aa C36:3	37.4	8.8	18.4	64.5
	PC aa C36:2	89.9	14.2	65.4	153
	PC aa C36:1	41.3	9.6	25.6	70
	PC aa C38:5	23.5	5.5	13.2	37
	PC aa C38:4	84	15.2	50.9	131
	PC aa C38:3	23	6.2	14.4	38
	PC aa C38:1	3	0.59	2.09	4.84
	PC aa C40:5	13	3.66	6.23	23.2
	PC aa C40:4	6.75	2.24	3.36	12
	PC aa C40:3	0.94	0.29	0.53	1.66
	PC aa C42:6	0.24	0.07	0.14	0.5
	PC aa C42:5	0.27	0.07	0.16	0.46
	PC aa C42:4	0.19	0.04	0.1	0.27
	PC aa C42:2	0.07	0.01	0.05	0.12
	PC aa C42:1	0.06	0.02	0.01	0.1
	PC aa C42:0	0.07	0.01	0.05	0.11
	PC ae C30:2	0.02	0.02	0.01	0.05
	PC ae C30:1	0.65	0.21	0.34	1.27
	PC ae C30:0	0.5	0.13	0.34	0.98
	PC ae C32:2	0.4	0.07	0.27	0.63
	PC ae C32:1	2.17	0.41	1.51	3.53
	PC ae C34:3	1.7	0.31	1.26	2.57
	PC ae C34:2	6.15	1.15	4.17	10.1
	PC ae C34:1	6.93	1.21	4.89	11.1
	PC ae C34:0	1.38	0.3	0.9	2.31
	PC ae C36:0	0.67	0.17	0.39	1.1
	PC ae C36:5	2.72	0.69	1.73	4.5
	PC ae C36:4	5.37	1.17	3.41	8.72
PC ae	PC ae C36:3	3.25	0.59	2.39	5.32
	PC ae C36:2	7.27	3.47	3.08	20.9
	PC ae C36:1	4.09	1.41	1.96	8.57
	PC ae C38:6	1.36	0.26	1.01	2.12
	PC ae C38:5	5.11	1.02	3.7	7.9
	PC ae C38:4	7.58	1.59	4.78	13.1
	PC ae C38:3	1.83	0.41	1.08	3.09
	PC ae C38:2	1.29	0.31	0.78	2.29
	PC ae C38:1	1.17	0.26	0.74	1.85
	PC ae C38:0	0.4	0.08	0.28	0.65
	PC ae C40:5	1.6	0.33	1.02	2.85
	PC ae C40:1	0.38	0.07	0.29	0.58
	PC ae C40:4	1.26	0.24	0.82	2.09

		PC ae C40:3	0.45	0.09	0.33	0.7
		PC ae C40:2	0.24	0.04	0.18	0.36
		PC ae C40:6	1.24	0.33	0.82	2.52
		PC ae C42:5	0.22	0.05	0.16	0.36
		PC ae C42:4	0.22	0.04	0.15	0.36
		PC ae C42:3	0.16	0.03	0.11	0.25
		PC ae C42:2	0.14	0.03	0.09	0.24
		PC ae C42:1	0.17	0.03	0.1	0.26
		PC ae C42:0	0.09	0.02	0.06	0.18
		PC ae C44:6	0.13	0.03	0.1	0.2
		PC ae C44:5	0.11	0.02	0.08	0.17
		PC ae C44:4	0.06	0.01	0.04	0.1
		PC ae C44:3	0.05	0.01	0.01	0.09
	Lyso	LysoPC a C14:0	1.26	0.26	0.84	1.95
		LysoPC a C16:1	1.4	0.29	0.79	1.95
		LysoPC a C16:0	29.2	4.54	19.8	45.9
		LysoPC a C17:0	0.77	0.39	0.26	2.35
		LysoPC a C18:2	16.2	4.39	9.1	30.9
		LysoPC a C18:1	12	2.29	8.6	18.5
		LysoPC a C18:0	14.5	2.87	8.41	24.9
		LysoPC a C20:4	5.97	1.15	3.62	9.1
		LysoPC a C20:3	1.39	0.61	0.02	3.07
		LysoPC a C24:0	0.1	0.02	0.07	0.16
		LysoPC a C26:1	0.09	0.02	0.06	0.13
		LysoPC a C26:0	0.17	0.04	0.03	0.29
		LysoPC a C28:1	0.17	0.04	0.11	0.28
		LysoPC a C28:0	0.24	0.05	0.17	0.4
Sterol lipids	CE	CE(14:0)	27.2	10.6	11.2	55.1
		CE(14:1)	1.06	1.97	0.05	12.9
		CE(15:0)	7.25	3.76	1.81	24.1
		CE(15:1)	1.28	2.17	0.02	14.6
		CE(16:0)	248	45	158	364
		CE(16:1)	184	93	64	517
		CE(17:0)	5.85	2.46	2.47	12.4
		CE(17:1)	13.9	5.5	5.9	30.8
		CE(18:0)	33	13.7	15.1	81.6
		CE(18:1)	772	297	371	1865
		CE(18:2)	1925	418	1295	3215
		CE(18:3)	103	26	57	182
		CE(20:0)	0.99	0.66	0.05	2.66
		CE(20:1)	1.52	0.81	0.3	4.14
		CE(20:3)	29.4	11.3	15.5	68.2
		CE(20:4)	435	150	231	889
		CE(20:5)	9.97	3.03	5.08	19.3
		CE(22:0)	0.53	0.43	0.05	1.67
		CE(22:1)	0.47	0.55	0.03	1.97

CE(22:2)	0.46	0.24	0.02	1.04
CE(22:5)	7.72	3.01	3.39	17.2
CE(22:6)	21.7	7.6	10.5	51.7

Treatments were: 1) Milk Replacer: commercial milk substitute rich in animal fat and coconut oil (CO); milk substitute rich in polar lipids (PO) or milk substitute rich in soy lipids (SO); 2) Diet: solid feed containing soy lipids (SD) or lipids from cow milk fat globular membranes (PD). Sphingolipids: ceramide (Cer); sphingomyelin (SM); sphingomyelin with a hydroxyl group (SM (OH)); ceramide with a hexose sugar residue attached to the sphingoid base and a fatty acid chain (HexCer); ceramide with two hexose sugar residues attached to the sphingoid base and a fatty acid chain (Hex2Cer); ceramide with three hexose sugar residues attached to the sphingoid base and a fatty acid chain (Hex3Cer). Glycerolipids: diacylglyceride (DG); triglyceride (TG). [1]Phospholipids containing choline: phosphatidylcholine with an acyl chain (PC aa); phosphatidylcholine with an acyl-alkyl chain (PC ae); lysophosphatidylcholine (Lyso). Sterol lipids: Cholesteryl ester (CE). n = 8 per treatment group; obtained from pooled samples coming from 3 piglets per pen.
